# Comparative analysis of plant *MKK* gene family reveals novel expansion mechanism of the members and sheds new light on functional conservation

**DOI:** 10.1186/s12864-018-4793-8

**Published:** 2018-05-29

**Authors:** Min Jiang, Zhaoqing Chu

**Affiliations:** 1 Shanghai Key Laboratory of Plant Functional Genomics and Resources, Shanghai Chenshan Botanical Garden, Shanghai, China; 2 Shanghai Chenshan Plant Science Research Center, Chinese Academy of Sciences, Shanghai, China

**Keywords:** MAPK kinase, Gene duplication, Evolution, Gene expression, NTF2 domain, Docking site

## Abstract

**Background:**

Mitogen-activated protein kinase (MAPK) cascades play critical functions in almost every aspect of plant growth and development, which regulates many physiological and biochemical processes. As a middle nodal point of the MAPK cascades, although evolutionary analysis of MKK from individual plant families had some reports, their evolutionary history in entire plants is still not clear.

**Results:**

To better understand the evolution and function of plant MKKs, we performed systematical molecular evolutionary analysis of the *MAPKK* gene family and also surveyed their gene organizations, sequence features and expression patterns in different subfamilies. Phylogenetic analysis showed that plant MAPKK fall into five different groups (Group A–E). Majority orthology groups seemed to be a single or low-copy genes in all plant species analyzed in Group B, C and D, whereas group A MKKs undergo several duplication events, generating multiple gene copies. Further analysis showed that these duplication events were on account of whole genome duplications (WGDs) in plants and the duplicate genes maybe have undergone functional divergence. We also found that group E MKKs had mutation with one change of serine or theronine might lead to inactivity originated through the ancient tandem duplicates in monocots. Moreover, we also identified MKK3 integrated NTF2 domain that might have gradually lost the cytoplasmic-nuclear trafficking activity, which suggests that they may involve with the gene function more and more sophistication in the evolutionary process. Moreover, expression analyses indicated that plant *MKK* genes play probable roles in UV-B signaling.

**Conclusion:**

In general, ancient gene and genome duplications are significantly conducive to the expansion of the plant *MKK* gene family. Our study reveals two distinct evolutionary patterns for plant MKK proteins and sheds new light on the functional evolution of this gene family.

**Electronic supplementary material:**

The online version of this article (10.1186/s12864-018-4793-8) contains supplementary material, which is available to authorized users.

## Background

Plants have evolved intricate mechanisms to respond various perturbations or stresses of external environment to maintain normal growth and development. Plants cannot move its position to avoid the adverse environments and can only adapt to the abiotic or biotic stresses in situ. Therefore, plants have developed a set of precise regulatory mechanisms to perceive the cues from environment, to transduce and amplify the signal, and to respond to stress at the molecular, cellular and physiological levels [1]. Protein modification is a primary mode of signal transduction such as phosphorylation. These compounds execute diverse signaling cascades in post-translational level by means of catalyzing the addition of phosphate groups to serine and threonine/tyrosine residues in their target proteins, which switch downstream stress-responsive genes on or off in both prokaryotic and eukaryotic cells [2]. A tripartite mitogen-activated protein kinase (MAPK) signaling cascade is sophisticated multienzyme complexes that are highly conserved in evolution and fundamental signaling transduction pathways that play important roles in stress resistance, hormonal responses and developmental regulation [3–5]. A canonical MAPK cascade is composed of three continuously acting protein kinases. The MAPKK kinase (MAPKKK or MEKK) phosphorylates and activates the MAPK kinase (MAPKK, MEK or MKK) on the S/T-X_3–5_-S/T motif which is located in their phosphorylation region. Subsequently, the activated MKK can phosphorylate MAPK (also called MPK) at their threonine and tyrosine (TXY) residues located in the MAPK activation loop [3, 6]. Moreover, scaffold proteins, shared docking domains and adaptor or anchoring proteins can mediate the formation and integrity of a specific MAPK cascade [7–9]. Numerous reports indicated that the MAPK cascades were connected with signaling pathways activated by many critical environmental factors, such as salinity, low temperature, drought, heavy metals, high pH, biotic stresses and plant hormone responses [5, 10].

With the completion of the genome sequence in a variety of plants, many members of MAPK cascades have been annotated. MAPK protein was originally identified in the model plant *Arabidopsis thaliana*. Subsequent comprehensive bioinformatics analyses of these protein families have identified in plant genomes, including *A. thaliana* [11], *Oryza sativa* [12], *Brassica napus* [13], apple [14], *Brachypodium distachyon* [15], and so on. However, these researches have been limited to one or a few plant with no change in this status. To date, knowledge about the function of MAPK cascade, especially MKKs that are the middle nodal point of the MAPK cascade, is rather limited in several species, including Arabidopsis*.* MKKs containing the least members which is only half of MAPKs may be able to activate more than one MAPK protein which indicates a large number of combinatorial possibilities to form specific MAPK cascade [16]. The 10 MKKs encoded in the Arabidopsis genome, among which one (MKK10) have one mutation in conserved S/T-X_5_-S/T motif, can be classified into four groups (A to D) based on their evolutionary relationship. MKK1 and MKK2 which belongs to group A in Arabidopsis can activate downstream MPK4 in responses to cold and salinity, and also mediate innate immunity responses [17]. Moreover, MKK1 also mediates H_2_O_2_ metabolism via MPK6-coupled signaling in Arabidopsis [18]. MKK6 was identified to activate MPK13 in mutant yeasts [19] and *NQK1* which is an MKK6 ortholog in tobacco was involved in the formation of the cell plate at the late M-phase of cytokinesis [20]. MKK3, the only member of group B MKK, which has a nuclear transfer factor 2 (NTF2) domain in their 3′ extension C- terminus region that mediates nuclear import of RanGDP [21], plays a pivotal role in jasmonate (JA)-mediated signal transduction pathway [22] and blue light-mediated signaling [23]. MKK4/5, group C *MKK* gene members, were revealed to regulate in stress responses [24] and ABA responses [25]. With the exception of MKK7 and MKK9, which were also reported be related to polar auxin transport and ethylene signaling pathways, respectively [26, 27], there is little information about the function of other group D MKKs, which may due to the low-level transcripts of them. Previously, it has been suggested that MKK10 had not biologically function because of the mutation in active site. However, recent report suggested that OsMKK10–2 plays a crucial role in salicylic-acid (SA) signal-mediated pathogen defense response [28].

So far, evolutionary history studies of MAPK cascades in plants have executed by using a limited number of plants species. Some studies used MAPK sequences from several representative plants had revealed a phylogeny covering > 800 million years of evolution [29] and showed novel activation loop variants [30]. Gene duplication is a basic source of new genes in evolution, providing novel opportunities for evolutionary success [31], while whole genome duplications (WGDs) is one of the important evolutionary feature. Evidence for WGDs has been detected at least one time in most plant taxa, while it has at least two rounds of WGDs that are assumed to have place approximately 60–70 and 23–43 Myr ago in *A. thaliana* [32, 33]. The results of WGDs are gene amplification double of all genes [31, 32]. Ever since a WGD event, plants take up removing most redundant gene copies in a long evolutionary process, but some duplicated copies are retained depending on their environmental adaptation [32, 34], which is the cause of varying numbers of multigene family members between plant species.

In contrast to MAPKs, MKK’s evolutionary across different plant species has not been reported. Fortunately, the sequence availability of plant genomes allowed us to investigate the evolutionary history of MKKs from different plant species. In this study, we have identified *MAPKK* genes, examined their structural information, and performed phylogenetic analyses from major plant lineages. Our analyses identify a comprehensive overview of the phylogenetics and showed that two distinct evolutionary patterns in different MKK groups, as well as functional evolution of MKK3 have distinct difference compared to those of other groups. Expression patterns of these genes are analyzed in five taxa with various tissues and treatments, and the potential function of these genes is discussed.

## Results

### Identification of *MAPKK* genes in 51 plant species based on the conserved S/T-X_5_-S/T domain

In order to investigate the diversity and evolution of MAPKK protein architectures across plants, identification of the *MAPKK* gene family was performed from 51 genome publicly available plants that contained the maximum number of species across the plant lineages starting from the unicellular eukaryote alga to the angiosperm *A. thaliana* (Table 1). We found that the numbers of MAPKKs varied from species to species across the entire plant kingdom. 365 non-redundant MKK sequences were retrieved in total (Table 1, Additional file 1). The tetraploid soybean genome contained the largest number *MAPKK* genes, whereas the lower alga plant *Volvox carteri* only contained one. In addition, *B. distachyon* (12), *Capsella rubella* (11), *Gossypium raimondii* (11), *Manihot esculenta* (11), *Populus trichocarpa* (11) and *Setaria italic* (12) contained higher number of MKKs (Table 1, Additional file 1).

**Table 1:** Table representing genome size of different plant species and number of *MAPKK* genes present per genome (species)

Group	Order	Species	Abbr.	Genome size (Mbs)	Copy number	Database*
Angiosperm	Brassicales	*Arabidopsis thaliana*	At	135	10	PLAZA
		*Brassica napus*	Bn	630	8	NCBI
		*Brassica oleracea*	Bo	630	10	Oil Crops Genomics Database
		*Brassica rapa*	Br	283.8	14	PLAZA
		*Capsella rubella*	Cru	134.8	11	PLAZA
		*Carica papaya*	Cp	135	9	PLAZA
		*Thellungiella parvula*	Tp	140	9	PLAZA
	Malvales	*Gossypium raimondii*	Gr	761.4	11	PLAZA
		*Theobroma cacao*	Tc	330.8	8	PLAZA
	Sapindales	*Citrus sinensis*	Cs	319	7	PLAZA
	Myrtales	*Eucalyptus grandis*	Eg	691	6	PLAZA
	Malpighiales	*Jatropha curcas*	Jc	410	10	Jatropha Genome Database
		*Manihot esculenta*	Me	760	11	PLAZA
		*Populus trichocarpa*	Pt	422.9	11	PLAZA
		*Ricinus communis*	Rc	400	6	PLAZA
	Fabales	*Glycine max*	Gm	975	14	PLAZA
		*Lotus japonicus*	Lj	472	7	PLAZA
		*Medicago truncatula*	Mt	257.6	6	PLAZA
		*Phaseolus vulgaris*	Pv	521.1	9	Phytozome
	Cucurbitales	*Citrullus lanatus*	Cl	425	6	PLAZA
		*Cucumis sativus*	Csa	203	6	Cucurbit Genomics Database
		*Cucumis melo*	Cm	375	6	PLAZA
	Rosales	*Fragaria vesca*	Fv	240	5	PLAZA
		*Malus domestica*	Md	881.3	9	PLAZA
		*Prunus persica*	Ppe	451.9	10	PLAZA
	Vitales	*Vitis vinifera*	Vv	487	5	PLAZA
	Solanales	*Capsicum annuum*	Ca	3480	5	Pepper Genome Database
		*Solanum lycopersicum*	Sl	900	5	PLAZA
		*Solanum melongena*	Sme	1093	4	Eggplant Genome DataBase
		*Solanum tuberosum*	St	800	5	PLAZA
	Ericales	*Actinidia chinensis*	Ac	616.1	9	Kiwifruit genome sequence
	Gentianales	*Coffea canephora*	Cc	710	8	Coffee Genome Hub
	Caryophyllales	*Beta vulgaris*	Bv	566.6	5	PLAZA
		*Dianthus caryophyllus*	Dc	622	8	Carnation DB
	Poales	*Brachypodium distachyon*	Bd	272	12	PLAZA
		*Hordeum vulgare*	Hv	5100	6	PLAZA
		*Musa acuminata*	Ma	472	9	PLAZA
		*Oryza sativa*	Os	372	8	PLAZA
		*Setaria italica*	Si	405.7	12	PLAZA
		*Sorghum bicolor*	Sb	697.5	7	PLAZA
		*Zea mays*	Zm	2500	8	PLAZA
	Amborellales	*Amborella trichopoda*	Atr	748	6	PLAZA
Gymnosperm	Coniferales	*Picea abies*	Pa	1960	7	Spruce genome project
Pteridophyte	Selaginellales	*Selaginella moellendorffii*	Sm	212.5	3	PLAZA
Bryophyte	Funariales	*Physcomitrella patens*	Pp	480	7	PLAZA
Algae	Chlamydomonadales	*Chlamydomonas reinhardtii*	Cr	130	2	PLAZA
		*Volvox carteri*	Vc	125.4	1	PLAZA
	Mamiellales	*Micromonas pusilla*	Mp	22	2	PLAZA
		*Ostreococcus lucimarinus*	Ol	13.2	1	PLAZA
	Naviculales	*Phaeodactylum tricornutum*	Pti	27.4	1	PLAZA
	Laminariales	*Saccharina japonica*	Sj	537	2	Saccharina Genome Project [72]
Vertebrates	Primates	*Homo sapiens*	Hs	2996.43	7	UCSC Human Gene
Fugi	Saccharomycetales	*Saccharomyces cerevisiae*	Sc	12.1249	4	SGD
	Schizosaccharomycetales	*Schizosaccharomyces pombe*	Sp	12.5913	3	PomBase

*Database websites: PLAZA, http://bioinformatics.psb.ugent.be/plaza/; NCBI, https://www.ncbi.nlm.nih.gov/; Phytozome, https://phytozome.jgi.doe.gov/pz/; Pepper Genome Database, http://peppersequence.genomics.cn/page/species/index.jsp; Jatropha Genome Database, http://www.kazusa.or.jp/jatropha/; Cucurbit Genomics Database, http://cucurbitgenomics.org/; BRAD, http://brassicadb.org/brad/index.php; Kiwifruit genome Database, http://bioinfo.bti.cornell.edu/cgi-bin/kiwi/home.cgi; Eggplant Genome DataBase, http://eggplant.kazusa.or.jp/; Coffee Genome Hub, http://coffee-genome.org/; Carnation DB, http://carnation.kazusa.or.jp/; Spruce genome project, http://congenie.org/citation; SGD, http://www.yeastgenome.org/; UCSC Human Gene, http://genome.ucsc.edu/; PomBase, http://www.pombase.org/

### Phylogenetic classification of MAPKKs into five subfamilies

**Fig. 1:**
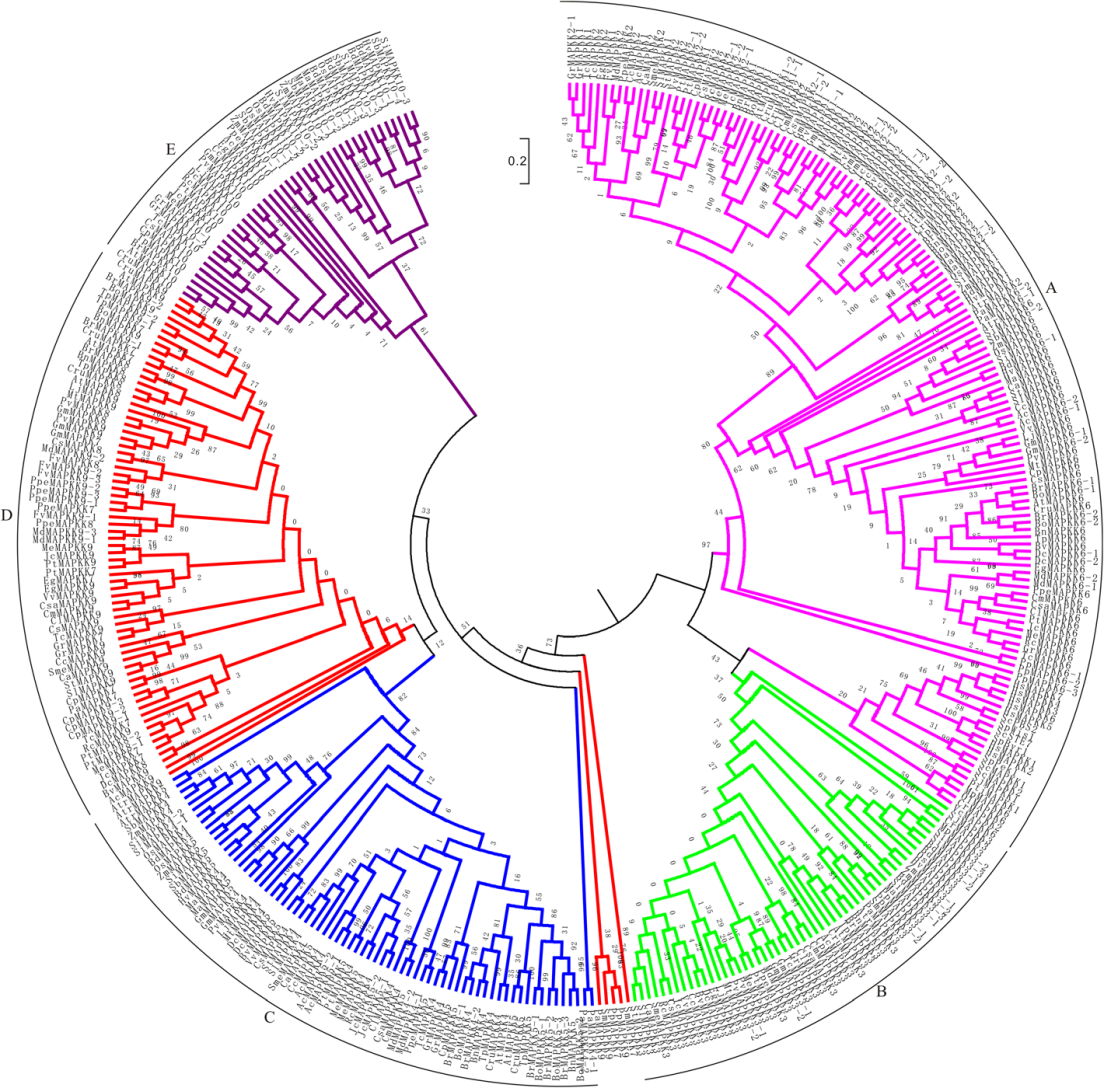
Maximum Likelihood phylogenetic trees of all plant MAPKKs. Phylogenetic analysis was carried with protein sequences for 365 MAPKK proteins from 51 plant species identified in this study

In order to elucidate the evolution history of plant MAPKK proteins, comprehensive analyzing the phylogeny of all identified 365 protein sequences from 51 sequenced plant species to date plus the MKKs of *Homo sapiens* and two yeasts were performed using maximum likelihood (ML) methods. According to phylogenetic analyses and subcellular localization prediction results, the plant MAPKKs can be divided into five main clades and designated as A, B, C, D and E, respectively (Fig. 1; Additional file 1), which is inconsistent with what was reported for Arabidopsis and rice MKKs. The group A, B and C contains MKK1/2/6, MKK3 and MKK4/5, respectively. It is noteworthy that the MKK7/8/9/10 sequences were previously reported to belong to group D, whereas our results suggested that these subfamilies should be divided into group D (MKK7/8/9; OG5_150257) and E (MKK10; OG5_211998) in the study according to phylogenetic analyses with the addition of the Orthomcl analyses (Additional file 2). Moreover, six MKKs of lower algae plants including CrMKK2, MpMKK6, OlMKK6, PtiMKK1, SjMKK1 and VcMKK6 with human and yeasts plus group B MKKs were on the same evolutionary branch, suggested that ancient MKKs have existed before the split of animal and plant (Fig. 1). Although the six algae MKKs were not on the same evolutionary branch with the group A MKKs, they were highly homologous with their corresponding Arabidopsis MKKs. So, we still named them using the orthologous based on the names of Arabidopsis, such as CrMAPKK2. We defined 31 (6, 5, 7, 7 and 6) orthology groups in the five subfamilies (group A, B, C, D and E), respectively. Interestingly, 2 groups (A and B) had genes start from chlorophytes, indicating that more than one *MAPKK* gene might have occurred prior to the split of green algae and land plants. Nevertheless, group C and D had genes from gymnospermae and moss, respectively. In group D, with members from land plants including mosses, ferns, gymnosperms and eudicots, whereas without monocots, which suggested that it was lost the divergence between monocots and dicots. While it only contained members of group C in gymnosperms and angiosperms, implying that they originated in the common ancestor of spermophyte. Furthermore, most orthology groups in each species maintained single copy genes in group B MKKs.

### Common conserved domain compositions and genomic analysis within plant MAPKK groups

**Fig. 2:**
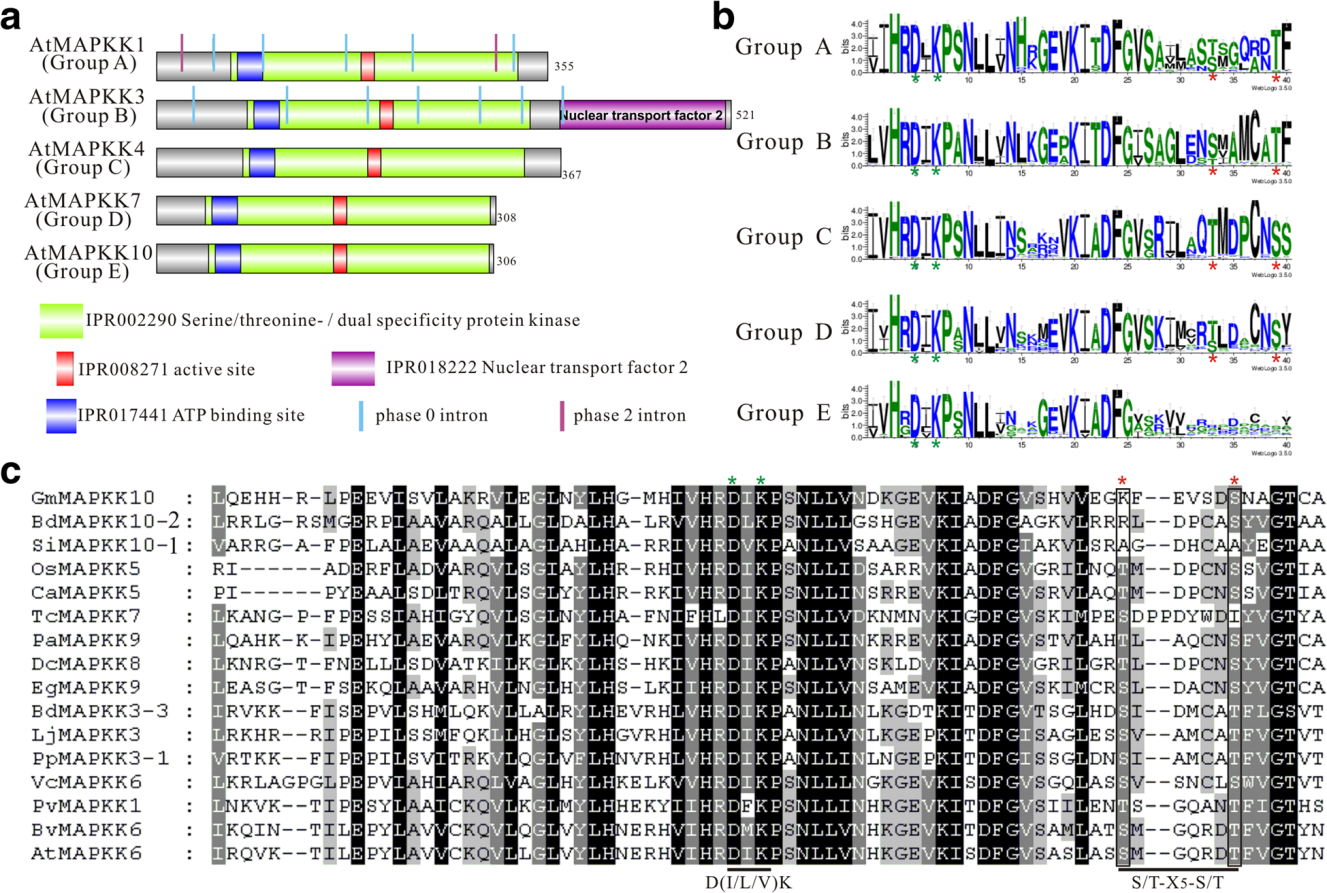
Gene structure and sequence features of conserved *MAPKK* genes. (**a**) Gene structure and protein motif. The structure of an *A.thaliana* gene (indicated on the left) is shown as an example for each group (in parenthesis on the left). Protein motifs are shown as colored boxes, whereas introns of different phase are shown as colored vertical lines. Protein motif architectures of the full-length proteins were drawn based on a search of InterPro program. IPR002290 indicates protein kinase motif, IPR017441 and IPR008271 means the ATP binding site and active site, respectively. The exons are drawn to scale. (**b**) Sequence features shown in the form of web logos representing the conserved S/T-X_5_-S/T motif and active site D(I/L/V)K motif of each group. The red and green stars indicate residues of functional or structural importance based on phylogenetic conservations. Logos were generated using the Weblogo3 application (http://weblogo.threeplusone.com/). (**c**) Multiple-sequence alignment of conserved S/T-X_5_-S/T motif and active site D(I/L/V)K motif portion of plant MAPKKs. The green star shows the active site and the red star indicate the phosphorylation site of MAPKK proteins in different plant species. Species information can be found in Additional file 1

To get a better overview of the characteristics of different plant MAPKKs, we further analyzed their sequences features. As expected, our motif organization analyses using InterProScan database revealed that putative plant MKK proteins contained common motifs in the same group, suggestive of functional similarities within each group. As illustrated in Fig. 2a, all of groups contain ATP binding site (IPR017441), protein kinase domain (IPR002290) and active site (IPR008271). Interestingly, group B MKKs have domain of Nuclear transport factor 2 (NTF2; IPR018222) near the carboxyl terminus, which is involved in the nuclear import of cargo proteins. These data provided us with clues for potential function of MKK proteins, including the idea that group B (MKK3) protein might participate in cytoplasmic-nuclear trafficking.

In order to understand the possible gene structural relationship among MKK orthologues and paralogues, we also analyzed the exon/intron organizations of different *MAPKK* genes using GSDS software. Within each group, most members displayed a similar exon/intron organization in the aspect of intron number, exon length, and intron phase. Notably, more similarities were observed in conserved regions such as protein kinase domain and active site (Fig. 2a). The numbers of intron also varied from 0 to 16 for another among different groups with wide divergence (Fig. 2a). Group A MKKs possesses introns ranged from 4 (*DcMAPKK6–2* and *PaMAPKK6–2*) to 14 (*AtrMAPKK2*) and dominated with 7 (60%), while the numbers of intron in group B MKKs ranged from 2 (*PaMAPKK3*) to 16 (*AtrMAPKK3*) and 8 occupied the main points (68%; Additional files 1 and 3). Remarkably, most groups C, D and E MKKs contain intronless (Fig. 2a, Additional files 1 and 3).

Next, we further examined the molecular weights (Mw) and isoelectric point (pI) of different MAPKK proteins using the online version of Compute pI/Mw tool. The Mw of MAPKK proteins varied from 11.276 (FvMKK8) to 146.588 (TcMKK2) kDa and the pI varied from 5.06 (ZmMKK3–2) to 10.15 (VvMKK5) (Additional file 4). The pI of groups D and E MKKs were in the basic ranges, while those of group A and group B MKKs were ranges from acidic to slightly acidic and group C MKKs were resided within slight alkaline. The average amino acid composition of MAPKK proteins were ranges from 0.85 (tryptophan) to 10.38 (leucine) (Additional file 5). Remarkably, the average abundance of the most important amino acids serine and threonine (S/T-X_5_-S/T) which were involved in the phosphorylation function of MKKs were 8.70 and 4.16, respectively. In addition, the average abundance of the hydrophobic amino acids in MAPKKs were relatively higher than ones of other amino acids such as alanine (6.51), isoleucine (5.85), leucine (10.38), proline (6.35) and valine (6.35) (Additional file 5).

The formerly studies have identified several consensus sequences that is associated with the roles of structure or function, including the S/T-X_5_-S/T motif in the activation loop [24], the docking site (K/R_2-3_X_1–5_L/IXL/I) in N-terminal domain [35, 36], the GxGxxGxV motif in the nucleotide binding domain (NB domain) and HK-X_6_-ALK motif in the ATP binding site [37], and D(I/L/V)K motif in active site [38]. Specifically, the serine or threonine in S/T-X_5_-S/T motif as the phosphorylation site play pivotal role in signal transduction pathway. In order to further identify consensus sequences that might be characteristic of phylogenetic group, we then performed sequence logos motif analyses using WebLogo 3 online tool for each group as an illustration of the sequences of ATP binding site and phosphorylation site of the protein kinase domain (Fig. 2b; Additional file 6). For examples, Fig. 2b showed the sequence information of active site D(I/L/V)K motif and phosphorylation site S/T-X_5_-S/T motif from each group. The stars indicated residues were important to the function of MAPKK. In all groups, the active site D(I/L/V)K motif were highly conserved despite occasional variations which contained novel active site, for instance, DFK (MdMAPKK2 and FvMAPKK1) or DMK (BvMAPKK6), which suggested that most MAPKK have the kinase activity. Furthermore, we observed an atypical phosphorylation site S/T-X_5_-S/T motif in group E MAPKK proteins, suggesting that these MAPKK may not have biologically function because the prior viewpoint that a mutation resulting in a change of serine or theronine led to the abolishment of kinase activity (Fig. 2c; [10, 37]). Hence, most group E MAPKKs (MKK10) maybe constitute a new group of inactive MAPKKs, although experimental tests for kinase activity need to be further validated. It was noteworthy that group E MAPKKs only existed in angiosperm plants, especially in monocots that have obviously gene expansion. In addition, plant MKKs posseed a highly conserved N-terminal extension which displayed a assumed MAPK docking site (D-site) K/R-K/R-K/RX_(1–5)_L/I-X-L/I [39]. During MAPK signaling, the existence of short D-site that is used in common complementary region for recognition on the MAPK promotes the ability of MKKs to recognize their cognate MAPKs. The MKKs contained group specific conserved docking-sites, K/R-K-K-X_1–5_-L-K-L/V (group A), L-K/R-K-K-L-X-P-L (group B), R-X-R-R/K-R-X_2_-L-X-L-X-L (group C), R-X-R-R-X_1–3_-L-X-L (group D) and R-X-R-R-X_1–4_-L-X-L/I (group E), respectively (Additional file 7). The presence of group specific D-sites in MAPKKs suggested that different MAPKK targets were group specific in spite of those of group D and E MKKs had unusual similarity that might be involved in the more close relationship of their evolution. So we can obtain the consequence that evolution of different MAPKKs were orthologous based on group specific.

### Gene number variation of the *MKK* gene family

**Fig. 3:**
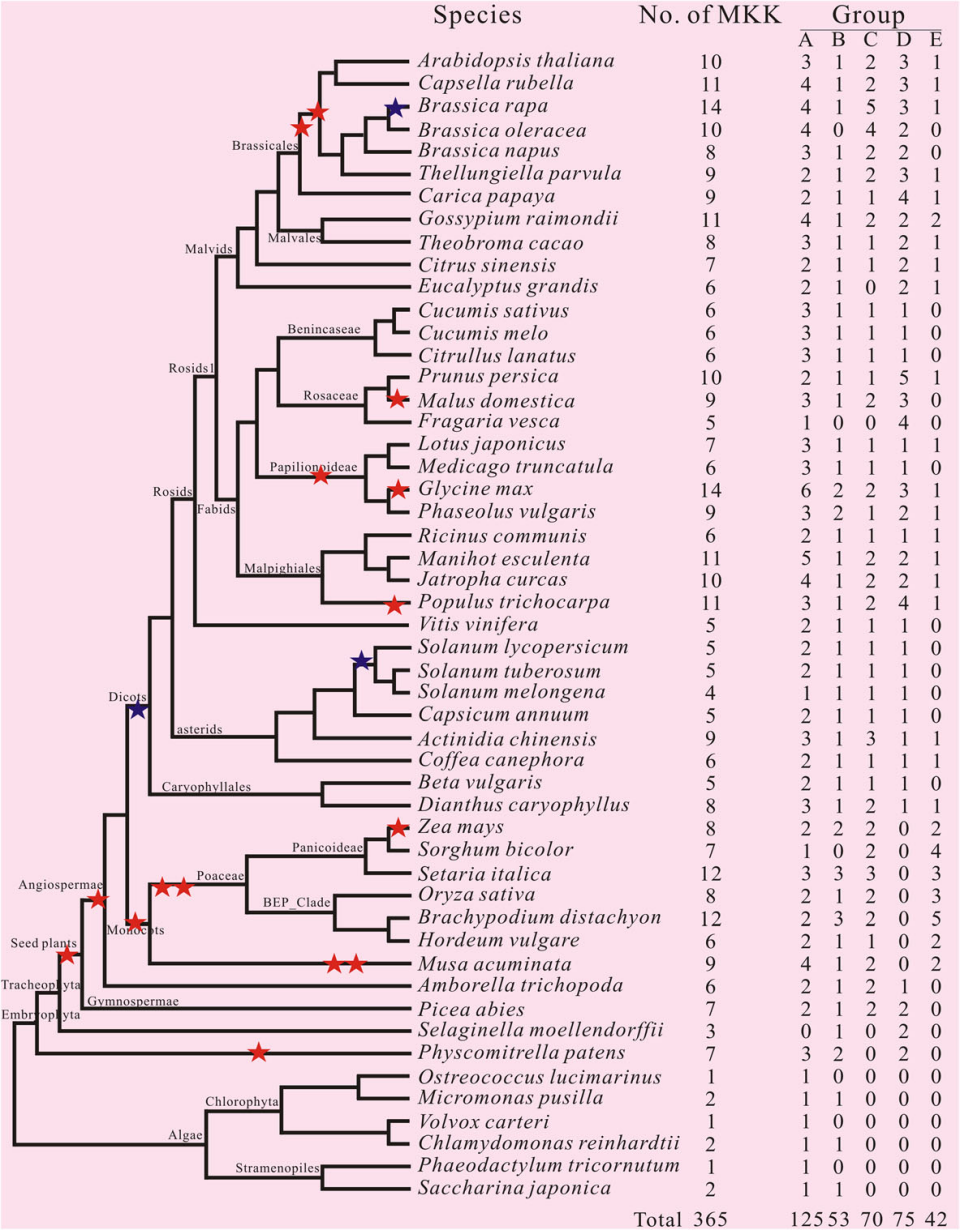
Phylogenetic relationships between the 51 plant species investigated in this study. The total number of MAPKK proteins and that of each groups identified in each plant genome is indicated on the right. The phylogenetic tree is modified from Phytozome (http://www.phytozome.net/). Red and blue stars indicate whole-genome duplication and triplication, respectively

The phylogenetic analyses showed that 125, 53, 70, 75 and 42 MAPKKs fall into group A, B, C, D, and E, respectively (Fig. 3). In non-seed plants, only nineteenth total *MKK* genes were identified, including three in *S. moellendorffii* and seven in *P. patens*, others in six different algae. In flower plants, the five *MKK* gene subfamilies were found in most species, except *B. oleracea*, *F. vesca* and *S. bicolor*, in which group B *MKK* genes were not detected, while group C *MKK* genes were not detected in *E. grandis* and *F. vesca*. The incomplete annotation of the genome sequence may be the reason for generating the result that group B or C *MKK* genes in this species has not discovered, because all other angiosperms have these genes. Except for MKK10 (group E) was only gathered in the angiosperm plants, the remaining MAPKKs were found in most species we surveyed. Our analyses showed that the canonical group D MKKs were absence in monocots, whereas the atypical MAPKK (group E) existed in both monocots and dicots. It was noteworthy that the number of group specific members in dicots is restricted to 0–2 genes, while monocots had strikingly large numbers, which might be associated with the WGDs of plant species in evolutionary process (Fig. 3).

Throughout the 51 complete genome sequences of plant species, the *MKK* gene numbers were greatly variable, ranging from one in the green alga *O. lucimarinus*, *P. tricornutum* and *V. carteri* to 14 copies in *B. rapa* and *G. max* (Table 1; Fig. 3). In many plant lineages, the increase in MAPKK proteins was involved in polyploidy or ancient polyploidization events (Fig. 3; [33]). For instance, there were 14 *MKK* genes in recently duplicated *G. max* genomes in which two rounds of WGDs have occurred. Similarly, the increase to identify the number of *MAPKK* genes in grasses was also probably attributable to three ancient polyploidization events in their evolutionary history [33]. A remarkable exception was maize (*Zea mays*), which possessed only 8 MAPKK proteins in spite of WGDs event has occurred. Nevertheless, these events were likely earlier resulting in loss of more duplicated genes (Fig. 3). An abnormally low number of *MKK* genes were identified in eggplant (*S. melongena*, 4 *MKK* genes) for which we can^,^t find explicit explanation. Further analysis showed that the copy number variation in plant species was mainly attributable to the difference in group A MKKs (Fig. 3), while the copy numbers were almost constant in other groups. For instance, only one copy was detected in most plants in group B and E MKKs (Fig. 3).

### Molecular evolutionary analyses

The genetic distance between groups D and C MKKs was 0.924, which was the lowest than other counterparts, indicated that there were higher sequence similarity between the group D and C MKKs (Table 2), which was consistent with their evolutionary history and Orthomcl analysis (Additional file 2). While group B MKKs showed the highest genetic distance to group E than to other groups, indicating that group B MKKs were less similar with group E than other MKKs in sequence level. On the contrary, the genetic distance between each group had not significant difference, indicating that the sequence divergence between each group MKKs were not significant. In addition, the average overall mean distance of MAPKK was 1.109 (standard error 0.0602).

**Table 2:** Genetic distance between different groups of MAPKKs calculated based on the amino acid sequences with the Jones–Taylor–Thornton (JTT) model

	A	B	C	D
A				
B	1.225			
C	1.202	1.365		
D	1.294	1.429	0.924	
E	1.452	1.628	1.111	0.996

To elucidate the evolutionary basis of functional diversification of each group MKKs, we have comprehensively evaluated the nonsynonymous-to-synonymous rates ratio (ώ = d*N*/d*S*) (Table 3) under different codon substitution-based evolutionary models. The mean ώ values were equally low for group B (ώ = 0.173) and group A (ώ = 0.228) MKKs, reflecting strong purify selection during the two groups MKK evolution. However, the mean ώ values of groups C, D and E MKKs were greater than 1, suggesting that the *MKK* genes are positive selection during the evolution process. Molecular evolutionary analysis revealed that group E and E^☆^ MKKs had roughly the same ώ values in spite of removing those of eudicots, indicating that positive selection were contributed by monocots during group E MKK evolution, while group A and B MKKs showed opposite results (Table 3). Moreover, in order to evaluate the difference between whole sequences and functional motifs, we also investigated the ώ values using the motif sequences (Additional file 8) under different codon substitution-based evolutionary models. The mean ώ values were equally low for all group MKKs when we used the sequences of D(I/L/V)K motif including S/T-X_5_-S/T motif, reflecting strong purify selection and indicating that D(I/L/V)K motif and S/T-X_5_-S/T motif are very conserved during the all group MKK evolution process (Additional file 8). Interestingly, except group C MKKs, it had similar results in the analyses of D-site or NB domain and ATP binding site (Additional file 8). In all, molecular evolutionary analyses using the motif sequences revealed that the difference of ώ values in different group MKKs were contributed by the sequences removing those of motifs which indicated that plant MKKs were very conserved, especially their functional domains.

**Table 3:** Molecular evolutionary analysis of the *MAPKK* genes using their whole cDNA sequences

Group	N	Ka	Ks	ώ	G + C content
A	125	0.17697	0.77494	0.228	0.442
A*	109	0.17783	0.76908	0.231	0.418
B	52	0.12819	0.73971	0.173	0.436
B*	42	0.13438	0.63808	0.211	0.437
C	69	0.33292	0.17082	1.949	0.563
C*	56	0.31983	0.23440	1.364	0.531
D	75	0.37734	0.10843	3.48	0.518
E	42	0.39775	0.28353	1.403	0.591
E^☆^	21	0.30844	0.22131	1.394	0.711

N, number of sequences; Ka, the number of nonsynonymous substitutions per nonsynonymous site; Ks, the number of synonymous substitutions per synonymous site; ώ, Ka/Ks. ^☆^ Sequences from dicots were excluded. *Sequences from monocots were excluded

### Multiple duplication events were identified in each group

The major force of the evolution of different species comes from gene duplication, which causes the gene to generate the gene families. In order to further understand the duplication and evolution events of the *MAPKK* genes, we also investigated the duplicated genes in plant genome from each orthology group (Additional file 9). The amount of duplicated genes represents the sizes of gene families, which are known as paralogs. Compared with the species with non duplicated genomes, the duplicated genome plants are more likely lead to duplicated *MAPKK* genes. Thus *G. max*, *B. rapa* and *B. distachyon* possess more duplicated genes. To further examine the evolution and duplication events of the group A *MAPKK* genes, we reconstructed phylogenetic trees using MEGA 6.0 with additional sequences from other plants. As shown in Fig. 3, all six representative green algae we surveyed contain one or two copy, but terrestrial plants such as moss *P. patens* have three or more copies, suggested that duplications likely took place after green algae moving towards land. As described above, group A *MKK* genes have more copies than other groups (Fig. 3). So, group A *MKK* genes can be further divided into six groups based on the phylogenetic relationship in land plants, namely subgroup A1, A2, A3, A4, A5 and A6 (Additional file 10). Of these six groups, subgroup A6 contained genes in moss, whereas subgroup A3 and A4 contained genes from monocots, and subgroup A1, A2 and A5 from eudicots. Within the subgroup A2-A5 clades, there were several independent duplications in grasses and dicots, respectively, indicating that the ancestor of corresponding lineage may have happened to the duplication events (Additional file 10).

**Fig. 4:**
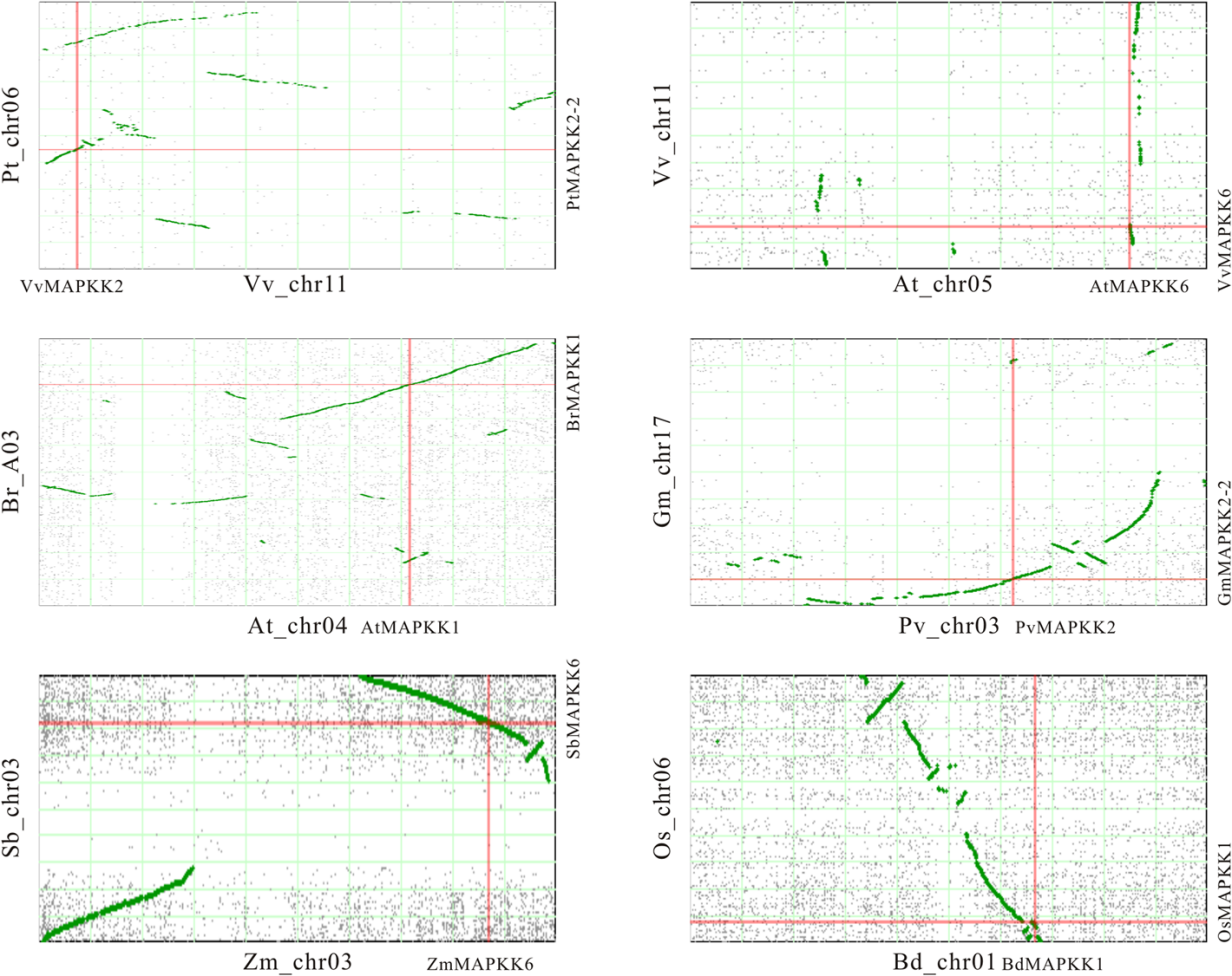
Examples of the detailed locations of representative pairs of genes duplicated in recent polyploidy events in the syntenic regions. At, *A. thaliana*; Bd, *B. distachyon*; Br, *B. rapa*; Gm, *G. max*; Os, *O. sativa*; Pt, *P. trichocarpa*; Pv, *P. vulgaris*; Sb, *S. bicolor*; Vv, *V. vinifera*; Zm, *Z. mays*; chr, chromosome

To explore the reason of these duplication events, we searched for the genomic regions containing the *MAPKK* genes which are possible synteny. Most duplicate gene pairs occurred in syntenic genomic regions, suggesting that these multiple gene copies were caused by whole genome or segmental duplications (Fig. 4; Additional files 11 and 12). Particularly, the duplication events of group A MKK1, MKK2 and MKK6 took place in the common ancestor of core eudicots corresponding with the γ WGD (Fig. 4; [40]). The two duplications of group A MKKs in Brassicaceae were involved in the α/β WGDs within the Brassicaceae lineage (Additional file 11; [40]). With the studies on the WGD events for many species were previously carried out, we collected these data and their paleopolyploidy histories (Fig. 3). We then assessed the duplication events to the influence of the size in group A MAPKKs. The comparison implied that the WGDs might be conducive to the expansion of the orthology groups from some species. In order to further confirm the roles of genome duplication to the gene family expansion, we carried out the co-relationship analysis between rounds of genome duplication about *MAPKK* genes. The correlation coefficient was calculated as 0.549 (*P* < 0.01) for the group A MAPKKs. The results suggested that the WGD significantly contributed to the gene expansion for group A MKKs.

**Fig. 5:**
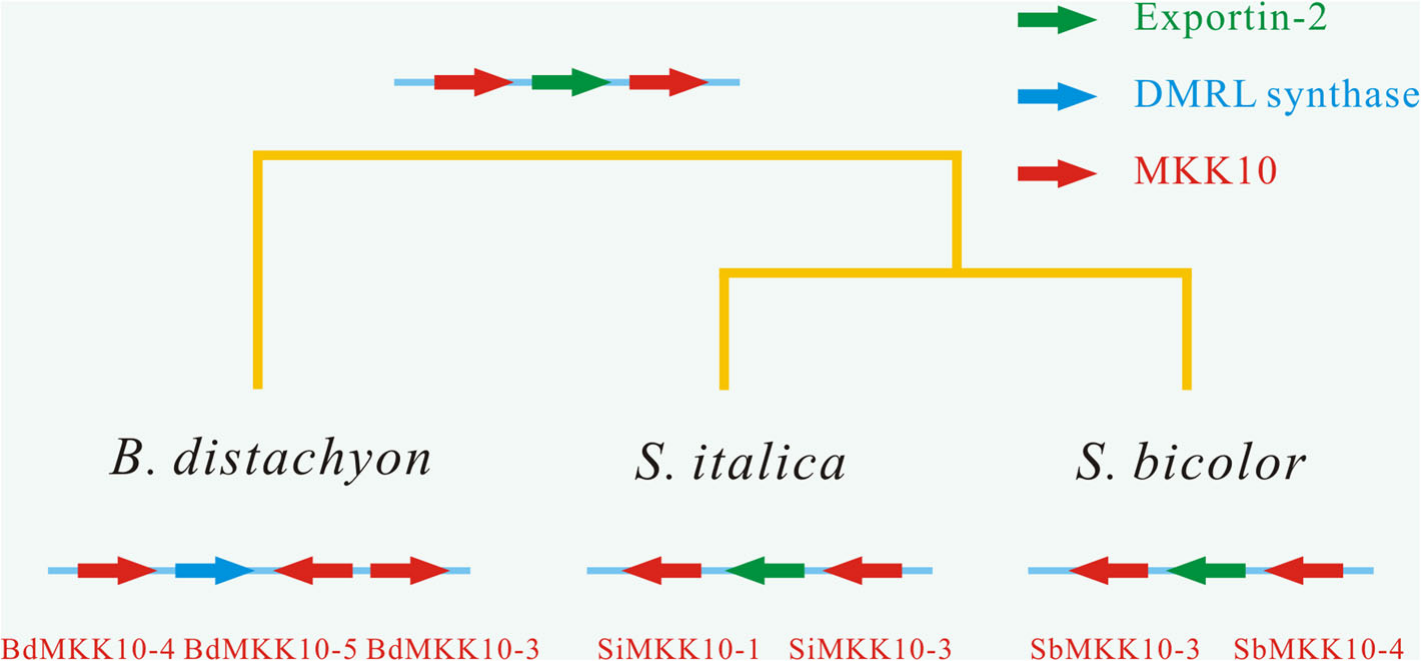
Evolutionary reconstruction analyses of the fate of an ancestral locus having Group E *MKK* genes in tandem position. Red arrowheads indicate *MKK* genes; Green arrowheads indicate *Exportin-2* genes; blue arrowheads indicate DMRL synthase genes

As mentioned above, group E *MKK* genes have a part mutation in the phosphorylation site S/T-X_5_-S/T motif from angiosperms. In angiosperm plants, group E MKKs can be further divided into six subgroups, namely subgroup E1-E6 (Additional file 13). Of these six subgroups, subgroup E1-E3 and E4-E6 contained genes from eudicots and monocots, respectively. Moreover, group D MKKs which was from land plants had not contain genes in monocots lineage. Group E MKKs displayed a conserved evolutionary pattern in eudicots with one gene copy, which was consistent with the state that these genes have a part of mutation in key active site. While the number of *MKK* genes in monocots prominently increased large members to 2–5 copies. Further syntenic analyses revealed that the monocots MKK10 multiple gene copies were caused by tandem duplication in the ancestor of corresponding lineage (Fig. 5; Additional file 14). The members of these duplications were often in the same orthology groups [41]. These duplicated genes were still in tandem in three species we analyzed and they were even multiplied during the segmental duplications of *B. distachyon*, *S. italica* and *S. bicolor*. Between the two MKK members, an *Exportin-2* gene was often found (Fig. 5). These results suggested that the tandem *MKK10* gene cluster in Poaceae originated in the common ancestral genomic contexts that result in modern monocot *MKK* genes.

Except group A and E MKKs, we also analyzed the phylogeny of other groups MKKs, respectively (Additional files 15, 16 and 17). Group C MKKs contained genes from seed plants, whereas group B MKKs contained genes from green plants. Group B MKKs showed one or low gene copy in all species that was a conserved evolutionary pattern. As a result, we can speculate that group B MKKs might have conserved and ancient functions that descended from the same ancestor of green plants. Even so, some recent duplication events also occurred. Within the group C and D MKKs, there was also one duplication event in Brassicaceae lineage (Additional files 16 and 17). Deeper syntenic analyses indicated that the group C MKK gene copies in Brassicaceae arise from WGD in the ancestor of this family (Additional file 18), while group D MKKs in Brassicaceae maybe contributed by segmental duplications or other forms.

### Expression profile of plant *MKK* genes indicates potential roles in flower tissues and UV-B signaling

**Fig. 6:**
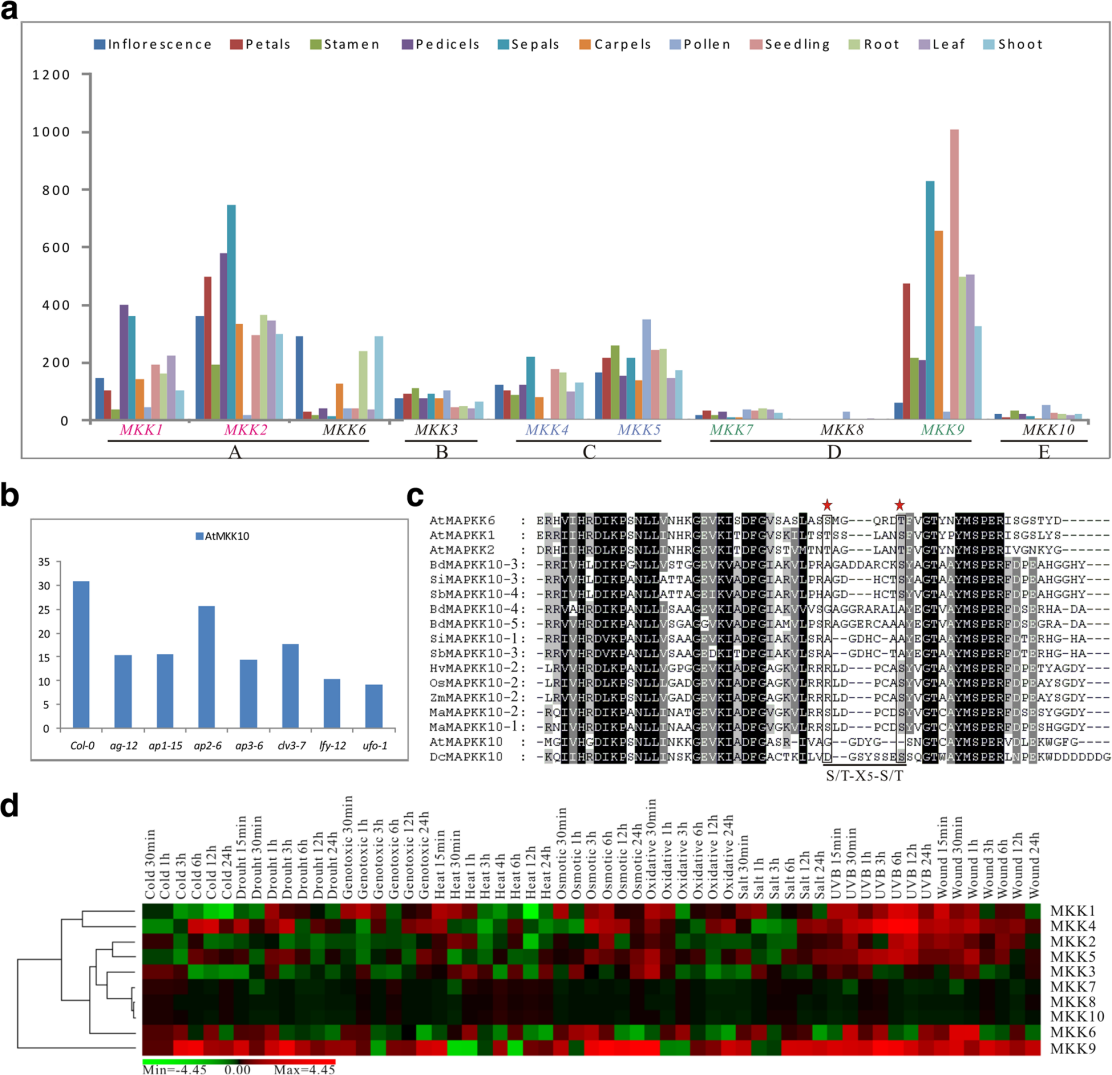
Expression and function analyses of Arabidopsis *MKK* genes. (**a**) Expression of Arabidopsis *MAPKK* genes. X-axis indicates representative tissues and developmental stages and Y-axis represents RPKM (reads per kilobase of mRNA length per million of mapped reads) value. Gene pairs resulted from recent duplications are shown in color. Duplicate genes in same color are paralogs from a recent duplication. (**b**) Expression of AtMKK10 in wild-type Columbia and *ag-12*, *ap1–15*, *ap2–6*, *ap3–6*, *clv3–7*, *lfy-12* and *ufo-1* mutants. The x-axis indicates different samples and y-axis indicates RPKM value. (**c**) A multiple sequence alignments of the key phosphorylation site of the MKK10 and other Arabidopsis Group A of MAPKK proteins. (**d**) Tiling array data of Arabidopsis MKK genes under drought, cold, high-salinity, heat, genotoxic, osmotic, oxidative and wounding and UV-B light treatment

A previous study showed that MKK8 and MKK10 have not expressed in shoot apices (including flower buds), mesophyll cells, mature leaves [26] and flowers (including developing young siliques) [42]. Based on the expression data of Arabidopsis from PLEXdb (AT40), we found that all Arabidopsis *MKK* genes are expressed (Fig. 6a). Except *AtMKK8*, most *MKK* genes have transcribed in the flowering stage. With the exception of MKK10, the Arabidopsis MKKs have a consensus S/T-X_5_-S/T in their activation domain. Even MKK10 have an atypical S/T-X_5_-S/T motif show detectable expression, indicating that they possibly have a new function other than becoming silenced. It is interesting that the expression of AtMKK10 is much higher in pollen compared with other tissues. Arabidopsis MKKs genetically exhibit the crosstalk in a variety of signaling pathways, therefore, a single *mkk* mutant under normal growth conditions is very difficult to obtain a specific phenotype [43]. A previous study showed that the *ap1–15*, *ap2–6*, *ap3–6*, *ag-12*, *clv3–7*, *lfy-12* and *ufo-1* mutants involve with flower development, whereas AtMKK10 was down regulated in these mutants (Fig. 6b), consistent with their expression in the flower. CLV has a potential role in the regulation of meristem size and has little expressed in the center of the meristem [44, 45]. The *bam* (CLV homologs) mutant exhibited rather weak phenotypes and regulated the male gametophyte development, leading to almost completely sterile plants [45]. Therefore, AtMKK10 which is regulated by CLV proteins may participate in the process of pollen development (Fig. 6c).

In rice, all except *OsMKK10–1* and *OsMKK10–3* are expressed in four reproductive stages from the microarray data (Additional file 19). Moreover, all maize and poplar *MKK* genes are expressed in different tissues (Additional file 19). However, the transcriptional levels in *PtMKKs* have a great difference, while maize is not obvious (Additional file 19). To further validate the transcripts of *B. distachyon MKK* genes in different tissues, we tested five different tissues that were a part of Bd21 seedling, root, leaf, stem and young caryopsis, to analyze the tissue specific expression patterns of *MKK* genes (Additional file 20). The results revealed that all except *BdMKK10–4* are expressed in five different tissues and *BdMKK6* and *BdMKK10–2* have higher transcriptional levels in young caryopsis than other tissues (Additional file 20). It is interesting that previous study show that MKK10 may not be biologically functional on account of the lack part of the phosphorylation site, but OsMKK10–2 can mediate the response to pathogen defense. Not as expected, MPK6 can be phosphorylated by OsMKK10–2 which have the kinase activity [28]. Moreover, we surveyed the gene expression of MKK under abiotic stresses that public microarray data of Arabidopsis under wound, drought, genotoxic, cold, osmotic, high-salinity, heat, oxidative and UV-B treatment were collected (Fig. 6d). Many plant *MKK* genes are up-regulated upon UV-B treatment, including AtMKK4 and AtMKK5. These results indicated that plant *MKK* genes may be involved in UV-B related signaling. To validate the expression profile of plant *MKK* genes in UV-B signaling, we performed the RT-qPCR experiments on RNA collected from different time points of *B. distachyon* plants under UV-B treatments. The results revealed that many *MKK* genes are up-regulated upon UV-B treatment, including *BdMKK4* and *BdMKK10–5*, especially *BdMKK10–5* originally have low-level transcripts in *B. distachyon* tissue (Additional file 20). These results indicated that *BdMKK* genes maybe also respond the UV-B stress. In a word, we can speculate that plant *MKK* genes play potential roles in UV-B related signaling.

## Discussion

### Evolutionary history and gene duplication of the MAPKK in plant lineages

Our phylogenies (Fig. 1) showed that the *MAPKK* gene family undergone at least two ancient duplications that lead to the five subfamilies. There was at least one gene in group A and B before the split of the green alga and terrestrial plants, while the group D MKKs took place before the divergence of the land plants. Numerous lineage-specific duplicate genes in land plants were likely also caused by the WGDs or segmental duplications. The first duplication event resulted in the appearance of the group C *MKK* genes lineages gave arise to after the divergence of vascular plants. Subsequently, the second duplication event took place during the divergence of angiosperms which caused the appearance of group E *MKK* genes. After the separation of gymnosperms and angiosperms, deep duplications occurred at least once in each group (Fig. 3). In angiosperms, it has been reported that an ancient WGD event has produced multigene copies [33]. So we observed a lot of gene and/or genome duplications in group A *MKK* genes in angiosperms (Fig. 3 and Additional file 10), which is the same as to the patterns of *rhomboid* and *SET* gene families [46, 47]. In eudicots, most species possess 4–8 *MAPKK* genes, apart from a few exceptions above 10, such as *B. rapa*, *P. trichocarpa* and *G. max*. Compared with their counterpart species, it has been shown that these species have undergone extra or more recent WGDs (Fig. 3). For example, Brassica experienced an extra recent whole-genome triplication (WGT) event in comparison to *A. thaliana* in Brassicales [48], compliance with the principle of roughly twice as many *MAPKK* genes in *B. rapa* in comparison with the other species (Fig. 3). Similarly, WGDs maybe also conducive to the *MAPKK* gene diversification in *G. max* (Fig. 3; [49]).

### Nuclear transfer factor 2 integration into MKK3 occurred alone in several plant lineages

**Fig. 7:**
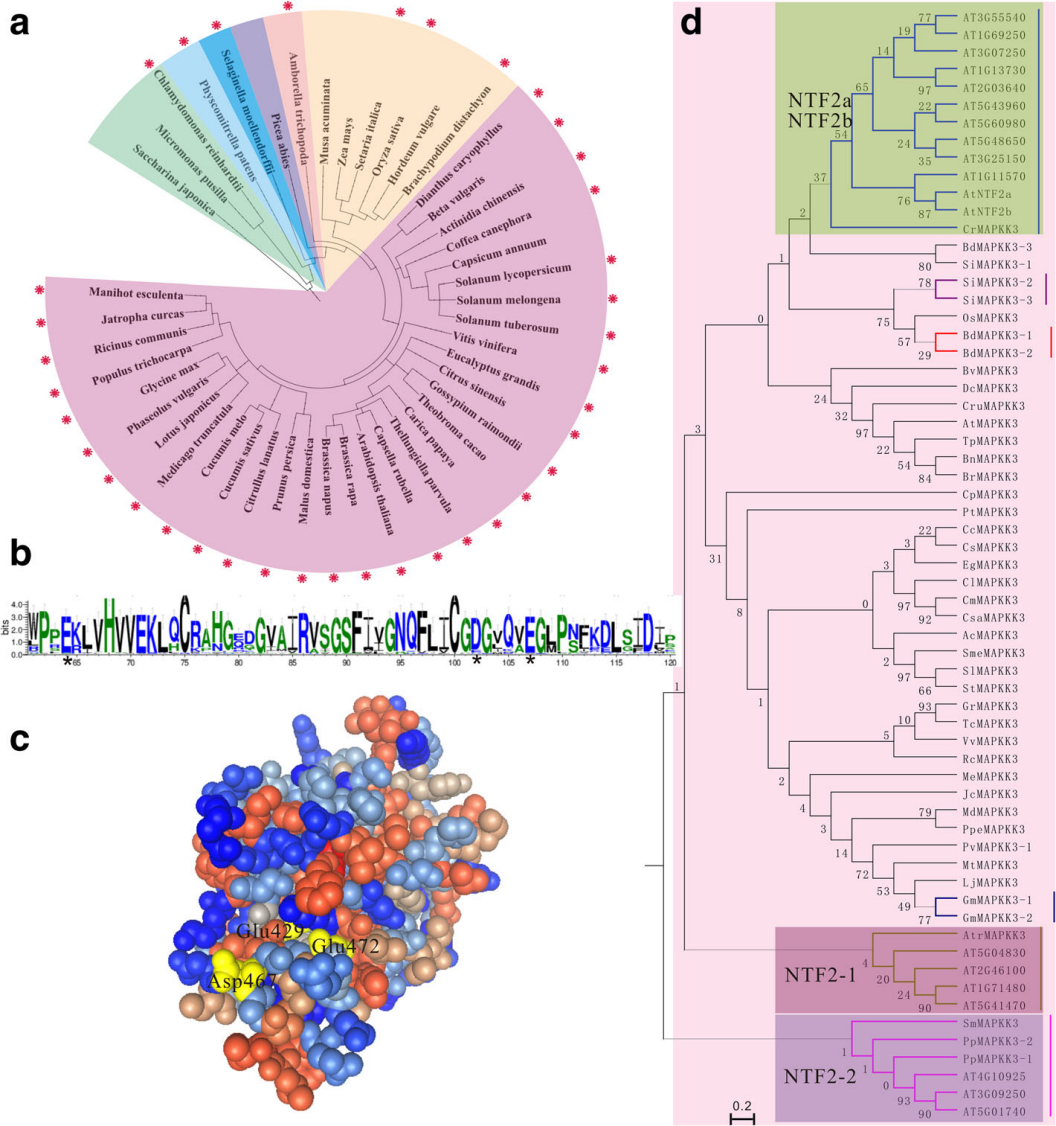
Evolutionary and structural analyses of the MKK3 fused NTF2 domain. (**a**) Visual representation of distribution of NTF2 fusions across plants. (**b** )Multiple sequence alignment logo of the NTF2 domains found in all MAPKK3 shows conserved core structural aspartic and glutamic residues that have been shown to be essential for binging the RanGDP. (**c**) Three-dimensional structural model of the NTF2 domain of an Arabidopsis MKK3 At5G40440 (aa 366–516) modeled after the best structural match, *S. cerevisiae* NTF2-like domain Mex67:Mtr2 (PDB: 4WWU). Conservation profile across all plant NTF2 found in MKK3 is overlapped on the structure, with critical aspartic and glutamic residues depicted in yellow. (**d**) Maximum likelihood phylogeny of all Arabidopsis NTF2 domain-containing proteins (black nodes) and the NTF2 detected as fusions in all plants (red nodes). Distinct Arabidopsis NTF2 clades that form fusions are highlighted. The names of species are given as in Additional file 1. Each duplicate gene pair was assigned a color and a line on the right

The NTF2 protein interacts specifically with the FxFG repeat-containing nucleoporins and Ran-GDP to form complexes [50] resulting in the cytoplasmic-nuclear trafficking of Ran [51]. The yeast (*Saccharomyces cerevisiae*) and *Caenorhabditis elegans* NTF2 orthology plays crucial roles and *ntf2* mutants have significant imperfection in nuclear trafficking [52]. However, the overexpression of *NTF2* also hampers the nuclear import of Ran protein in Arabidopsis [53]. Our data also showed that the NTF2 domain exists in 44 *MKK3* genes from 38 plant species as early in algae and in mosses, pteridophyta as well as seed plants, including previously reported *A. thaliana* (Fig. 7a; [53]). Then sequence alignment of NTF2 domain regions of all identified MKK3 exhibited a critical functional Ran-binding site such as aspartic and glutamic residues (Fig. 7b and c). Our results also revealed that the MKK-NTF fusions took place alone in several plants, including both eudicots and monocotyledons. In order to explore the evolutionary relationship all MKK3 fused NTF2 domain together with all NTF2 proteins from Arabidopsis, we performed the phylogenetic analyses which showed that fusions occurred at least three times and referred to homologs of NTF2a/b, NTF2–1 and NTF2–2 (Fig. 7d). In addition, the group B MKKs with NTF2 domain had a recent clades of lineage-specific paralogs, including one members such clades in soybean, *B. distachyon* and *S. italica*, respectively, revealing numerous gene duplication events (Fig. 7d). Apparently, some fusion proteins were more common in non-seed plants (i.e. NTF2a/b), while others were not extend to all phyla which may be involved in gene expansion in monocots and dicots (Fig. 7d). It has been suggested that most NTF2 domain in MKK3 have all the signatures of the functional NTF2, gradual loss of activity in the nuclear transport cannot be performed function as the MKK3 harboring NTF2 domain exhibit change of the conserved key residues, which may be involved with the gene function more and more sophisticated in the evolutionary process. Hence, the loss of NTF2 domain leads to sharply weaken the interaction ability of MKK3 [54]. It is interesting that the *Chlamydomonas* genome contains a MKK3 which possess the 3’-NTF domain, suggesting that this delusional arrangement has got through a long and successful evolutionary history in the photosynthetic eukaryotes lineage [10].

### Conservation of MAPKK protein kinase activities in spite of mutation

Although mutations have happened in some genes, new and inactive *MKK* genes were by no means generated as expected previous reports indicated that a mutation of MKKs in serine or theronine residues gave rise to the abolishment of these kinase activity [37]. However, depending on the public tiling array data and mutant phenotypes, apparently, the function of AtMKK10 that is catalytically inactive is associated with pollen development despite the sequence change in phosphorylation site (Fig. 6b, c). According to our RT-qPCR experiments, *BdMKK10–5* have same situation in its phosphorylation site is up-regulated after UV-B treatment (Additional file 20). More interesting, previous studies had shown that the average root growth rate for double mutant MKK10/MPK3 or MKK10/MPK6 was higher than anyone single mutant of them [55], and also the transcripts of MKK10 significantly changed after PPV-infected protoplasts [56]. More studied have been reported showed that part mutant *MAPKK* gene was involved with disease resistance in rice and ethylene-dependent cell death in maize, respectively, which can phosphorylate MAPK6 and MAPK3 in vivo [57, 58]. Furthermore, AtMKK10 have experienced mutations in phosphorylation key active site and exhibited different expression profiles from AtMKK7/8/9 (Fig. 6a). So, the mutant paralogs might still maintain the kinase activity to downstream target proteins.

## Conclusion

A total of 365 *MKK* genes were retrieved from 51 plant species using bioinformatics approaches based on the presence of a conserved S/T-X_5_-S/T domain. Our phylogenetic analyses revealed that group A and B MKKs first appeared in the common ancestors of all green plants, while group C and D MKKs were the rear to arise along with appearance of land or seed plants, respectively, with subsequent divergence of Group E MKKs in flowering plants. It was noteworthy that Group B MKKs were very different compared to those of other groups in many aspects of structure, including exon/intron organizations, phase pattern and NTF2 motif in the MKKs, as well as the biological functions of NTF2 domain with MKK3 will be gradually lost the activity during the evolution despite the loss of this domain maybe affected interaction capability of oneself. The group A MKKs expanded during the evolution, through WGDs followed by diversifications, while group E MKKs expanded during monocots evolution through the ancient tandem duplications. These data revealed novel viewpoints about the function and evolution history of plant MKKs.

## Methods

### Identification of *MAPKK* gene family members

Mitogen-activated protein kinase kinase (MAPKK) gene information from the model plant *A. thaliana* and rice were downloaded from The Arabidopsis Information Resources database (TAIR: http://www.arabidopsis.org/) [59] and the TIGR rice Genome Annotation Resources database (http://rice.plantbiology.msu.edu/) [60], respectively. To identify *MAPKK* genes of unknown species, BLASTP searches was conducted using orthologous protein sequences *A. thaliana* and *O. sativa* MAPKKs as the query search [61] in the publicly available PLAZA database (http://bioinformatics.psb.ugent.be/plaza/) [62] and phytozome database (http://www.phytozome.net/) [63]. The coding and genomic sequences of *MAPKK* genes were collected in 51 plant species (Table 1). The gene was only considered as probable *MAPKK* gene if it harbored the serine/threonine−/dual specificity protein kinase domain, including the active site motif D(L/I/V)K and the phosphorylation site S/T-X_5_-S/T within the activation loop, which was subsequently confirmed by scanning in InterPro software for the presence of MKK’s conserved domain [64]. All data were checked for redundancy and no any alternative splice variants were considered.

### Gene structure, sequence alignment, structural modeling and protein motif analyses

The exon/intron structure of individual *MKK* gene was confirmed by the Gene Structure Display Server (GSDS) software (http://gsds.cbi.pku.edu.cn/). Multiple sequence alignment of the identified amino acid sequences of *MAPKK* genes were performed by the Clustal Omega (http://www.ebi.ac.uk/Tools/msa/clustalo/). The alignment logos of the protein conserved domain were generated with WebLogo (http://weblogo.threeplusone.com/). The conserved domains and motifs in the MAPKKs were predicted using InterProScan against protein database (http://www.ebi.ac.uk/interpro/). The schematic of the structure of all members of MAPKKs were performed according to the InterProScan analysis results. Subcellular localization predictions of each MAPKK were carried out using CELLO server (http://cello.life.nctu.edu.tw/) [65]. The theoretical pI (isoelectric point) and Mw (molecular weight) of MAPKKs were performed using Compute pI/Mw tool online (http://web.expasy.org/compute_pi/). Structural modelling of the NTF2 domain was carried out with Phyre2 using amino acid sequence of the NTF2 domain from At5G40440 (aa 366–516) and the best structure (highest percent identity, most sequence coverage) modelled after *S. cerevisiae* NTF2-like domain Mex67:Mtr2 (PDB: 4WWU) was picked as a template [66].

### Molecular evolution analysis

The aligned cDNA sequences were used to calculate the value of Ka and Ks as well as their ratios using DNASP v5.10 [67]. To investigate the genetic divergence between each group, we counted the genetic distances on the basis of the amino acid sequences with the Jones-Taylor-Thornton (JTT) model in the Molecular Evolution Genetic Analysis 6.0 (MEGA 6.0) [68]. The overall mean distances of all MAPKKs were measured using MEGA6 software. The MEGA file used to construct phylogenetic tree were also devoted to calculate the overall mean distance of plant MAPKKs.

### Synteny and phylogenetic analyses

Phylogeny of all plant species we surveyed in this study was performed using PhyloT program (http://phylot.biobyte.de/) with the NCBI taxonomy IDs for each species and visualized with iTOL program. The phylogenetic trees were constructed based on the maximum-likelihood (ML) method with a JTT model and the bootstrap test was carried out with 2000 iterations by the MEGA 6.0 software [68]. To insure the more divergent domains could be conducive to the topology of the ML tree, all positions with 95% site coverage were eliminated. All the phylogenetic trees were deposited in Treebase (http://purl.org/phylo/treebase/phylows/study/TB2:S21358). The whole set of MAPKK protein sequences were assigned to different orthology groups using OrthoMCL [69] after Blastp all vs all (e-value 1e − 10) (http://orthomcl.org/orthomcl/). Synteny analyses of duplicate gene pairs and the WGD data were achieved from the Plant Genome Duplication Database (PGDD; (http://chibba.agtec.uga.edu/duplication/index/locus) [40]. MAPKKs we surveyed were produced by segmental duplication of the plant genome were determined by the analysis of the output file (Additional file 12) of the CoGe SynMap program (https://genomevolution.org/coge/SynMap.pl) obtained using a default parameter [70].

### Nomenclature of MAPKKs

In order to match the names with their function, all predicted MAPKKs were named using the orthologous based on the evolutionary relationship of MAPKKs with *A. thaliana* or *O. sativa* MKKs as suggested by Hamel et al. [10]. In the nomenclature systems, the first letter in upper case represented the corresponding genus name, the species name was shown in the second lowercase letter (in a few cases the first 1–2 letters), after which the MAPKK and number of corresponding orthologs of *O. sativa* or Arabidopsis with human and two yeast lineages, respectively. If more than one ortholog existed in individual species, that is paralogs, the second number is followed by a hyphen to distinguish between paralogs.

### Expression analysis

Expression data of Arabidopsis MAPKKs were obtained from PLEXdb (AT40), including following Arabidopsis tissues: inflorescence, petals, stamen, pedicels, sepals, carpels, pollen, seedling, root, leaf, shoot and pistil. As well as following Arabidopsis mutants: *ap1–15*, *ap2–6*, *ap3–6*, *ag-12*, *clv3–7*, *lfy-12* and *ufo-1*. Mapped reads were uniquely applied to deeper analysis. Gene expression levels were quantified by RPKM (reads per kilobase of mRNA length per million of mapped reads). Expression data of *MAPKK* genes in rice (GSE27726), poplar (GSE30507) and maize (GSE27004) were also downloaded from NCBI GEO database. Microarray data of Arabidopsis under abiotic treatment were also obtained from NCBI GEO data (GSE5620-GSE5628) in our analysis. For RT-qPCR analyses, 2-week-old *B. distachyon* Bd21 plants grown in each box were used for harvesting root, stem, leaf, young caryopsis and seedling samples. For UV-B treatments analysis, two-week-old *B. distachyon* Bd21 plants were treated as described previously [71]. Plants were harvested for further analyses after treatment 1 h, 3 h, 6 h, 12 h and 24 h, respectively. The primer-sets were listed in Additional file 21.

## Additional files

Additional file 1: **Table S1.** Table showing nomenclature gene name, locus ID, detailed genomic information and subcellular localization of plant MAPKKs. (PDF 1109 kb)

Additional file 2: **Table S2.** OrthoMCL automatic analysis about plant MAPKK. (TIF 40445 kb)

Additional file 3: **Fig. S1.** The exon/intron structures of plant *MAPKK* genes. (TIF 2182 kb)

Additional file 4: **Table S3.** Table representing molecular mass (in kDa) and isoelectric point of different *MAPKK* genes from 51 plant species identified during this study. (XLSX 53 kb)

Additional file 5: **Table S4.** Table shows average amino acid composition of plant MAPKKs. (TIF 2947 kb)

Additional file 6: **Fig. S2.** Weblogos represent the Nucleotide binding domain and the ATP binding site of each group. The stars indicate residues of functional or structural importance. (TIF 3413 kb)

Additional file 7: **Fig. S3.** Weblogos represent the docking site (D-site) of each group. The stars indicate residues of functional or structural importance. (TIF 17233 kb)

Additional file 8: **Table S5.** Molecular evolutionary analysis of the *MAPKK* genes in different motif. (TIF 22709 kb)

Additional file 9: **Table S6.** The duplicated gene pairs in the 51 plant genomes. (TIF 21703 kb)

Additional file 10: **Fig. S4.** Maximum Likelihood phylogenetic trees of plant group A MAPKKs. The red circle represents duplication events. (TIF 13036 kb)

Additional file 11: **Fig. S5.** Syntenic proofs of Group A MAPKKs in Brassicaceae . (TIF 15972 kb)

Additional file 12: **Table S7.** List of duplicated genes assigned to segmental duplication in plant genome, as detected by CoGe SynMap analysis. (XLSX 26 kb)

Additional file 13: **Fig. S6.** Maximum Likelihood phylogenetic trees of plant group E MAPKKs. The red circle represents duplication events. (TIF 3291 kb)

Additional file 14: **Fig. S7.** Syntenic proofs of Group E MAPKKs in monocots. (DOC 51 kb)

Additional file 15: **Fig. S8.** Maximum Likelihood phylogenetic trees of plant group B MAPKKs. (PDF 1039 kb)

Additional file 16: **Fig. S9.** Maximum Likelihood phylogenetic trees of plant group C MAPKKs. The red circle represents duplication events. (PDF 685 kb)

Additional file 17: **Fig. S10.** Maximum Likelihood phylogenetic trees of plant group D MAPKKs. The red circle represents duplication events. (PDF 148 kb)

Additional file 18: **Fig. S11.** Syntenic proofs of plant Group C MAPKKs. (TIF 2692 kb)

Additional file 19: **Fig. S12.** Expression profiles of other plant *MAPKK* genes. Y-axis represents RPKM value. The expression data were downloaded from rice (GSE27726), poplar (GSE30507) and maize (GSE27004) oligonucleotide array database, respectively. (TIF 7561 kb)

Additional file 20: **Fig. S13.** Expression profiles of *B. distachyon MAPKK* genes in different tissues and UV-B signaling. (DOCX 15 kb)

Additional file 21: **Table S8.** A lists of the RT-qPCR primers for the *MAPKK* genes of *B. distachyon*. (XLSX 16 kb)

## Abbreviations

D-site: Docking site
GSDS: Gene Structure Display Server
JTT: Jones-Taylor-Thornton
MAPK: Mitogen-activated protein kinase
MAPKK (MKK): Mitogen-activated protein kinase kinase.
MAPKKK (MEKK): MAPKK kinase.
ML: Maximum likelihood.
Mw: Molecular weights.
NB domian: Nucleotide binding domain
NTF2: Nuclear transfer factor 2.
PGDD: Plant Genome Duplication Database.
pI: isoelectric point.
RPKM: Reads per kilobase per million mapped reads.
RT-qPCR: Real-time quantitative polymerase chain reaction.
WGDs: Whole genome duplications.
WGT: Whole-genome triplication.
