# Supplementary material for: Comparative analysis of plant MKK gene family reveals novel expansion mechanism of the members and sheds new light on functional conservation
Source: BMC Genomics. 2018 May 29;19:407. doi: 10.1186/s12864-018-4793-8 (PMC5975520; doi:10.1186/s12864-018-4793-8)
Supplement: Supplementary file 1 — Table S1. Table showing nomenclature gene name, locus ID, detailed genomic information and subcellular localization of plant MAPKKs. (PDF 1109 kb) [file 12864_2018_4793_MOESM1_ESM.pdf]

Table S1 Table showing nomenclatured gene name locus ID detailed genomic information and subcellular localization of plant MAPKKs.

**Gene Name**	**Locus ID**	**Orientation**	**ORF**	**No. of a.a**	**No.of introns**	**5'-3' Coordinate**	**Subcellular localization**
Actinidia chinensis							
AcMAPKK1	Achn156001	+	1104	368	7	21976213-21979855	Extracellular
AcMAPKK3	Achn118561	-	1557	519	8	13588372-13594146	Cytoplasmic
AcMAPKK4	Achn226251	+	1011	337	0	279614-280624	Nuclear
AcMAPKK5-1	Achn164271	-	1077	359	0	15253782-15254858	Nuclear
AcMAPKK5-2	Achn301271	-	1083	361	0	10852855-10853937	Nuclear
AcMAPKK6-1	Achn069811	-	837	279	6	11180634-11186187	Nuclear
AcMAPKK6-2	Achn358101	-	1365	455	9	97916528-97929477	Nuclear
AcMAPKK9	Achn036041	+	954	318	0	2497903-2498856	Mitochondrial
AcMAPKK10	Achn313401	+	1038	346	0	16629594-16630631	Mitochondrial
Amborella trichopoda							
AtrMAPKK2	ATR_00146G00450	-	1065	355	14	856997-863481	Cytoplasmic
AtrMAPKK3	ATR_00083G00340	-	1554	518	16	1796077-1811154	Cytoplasmic
AtrMAPKK4	ATR_00011G01230	-	1038	346	0	5236608-5237645	Nuclear
AtrMAPKK5	ATR_00011G01240	-	1026	342	0	5286335-5287360	Nuclear
AtrMAPKK6	ATR_00056G01260	-	942	314	10	2356558-2364061	Cytoplasmic
AtrMAPKK9	ATR_00176G00020	-	1230	410	0	49215-50444	Nuclear
Arabidopsis thaliana							
AtMAPKK1	At4G26070	+	1065	355	7	13217797-13219695	Cytoplasmic
AtMAPKK2	At4G29810	-	1092	364	7	14593299-14595241	Mitochondrial
AtMAPKK3	At5G40440	+	1563	521	7	16182149-16184513	Extracellular
AtMAPKK4	At1G51660	+	1101	367	0	19154575-19155675	Nuclear
AtMAPKK5	At3G21220	+	1047	349	0	7445917-7446963	Nuclear
AtMAPKK6	At5G56580	-	1071	357	7	22904851-22906620	Nuclear
AtMAPKK7	At1G18350	+	924	308	0	6315686-6316609	Cytoplasmic
AtMAPKK8	At3G06230	+	882	294	0	1885496-1886377	Nuclear
AtMAPKK9	At1G73500	-	933	311	0	27639419-27640351	Cytoplasmic
AtMAPKK10	At1G32320	+	918	306	0	11655156-11656073	Cytoplasmic
Beta vulgaris							
BvMAPKK2	BV0G78470	-	1062	354	7	854050-865226	Nuclear
BvMAPKK3	BV4G01230	-	1569	523	8	1647195-1652007	Cytoplasmic
BvMAPKK5	BV8G13900	+	1116	372	0	2793375-2794490	Nuclear
BvMAPKK6	BV8G05680	-	1065	355	7	5941169-5945314	Nuclear
BvMAPKK9	BV3G06820	+	969	323	0	1546490-1547458	Nuclear
Brachypodium distachyon							
BdMAPKK1	Bradi1g51000	-	1047	349	7	49443017 - 49446304	Nuclear
BdMAPKK3-1	Bradi4g39490	-	1572	524	8	44107411 - 44111847	Cytoplasmic
BdMAPKK3-2	Bradi1g41860	+	1572	524	8	38580168 - 38590500	Nuclear

BdMAPKK3-3	Bradi3g11260	+	1704	568	8	9689290 - 9697559	Nuclear
BdMAPKK4	Bradi3g53650	-	1074	358	0	54258852 - 54260320	Nuclear
BdMAPKK5	Bradi1g46880	+	1032	344	0	45433080 - 45434736	Nuclear
BdMAPKK6	Bradi1g75150	-	1071	357	7	72118481 - 72123498	Cytoplasmic
BdMAPKK10-1	Bradi1g11525	+	1044	348	0	8573765 - 8574808	Nuclear
BdMAPKK10-2	Bradi1g69400	-	1023	341	0	67832493 - 67833815	Nuclear
BdMAPKK10-3	Bradi1g10800	-	1035	345	0	7865287 - 7866321	Nuclear
BdMAPKK10-4	Bradi1g10770	-	1026	342	0	7809375 - 7810400	Nuclear
BdMAPKK10-5	Bradi1g10790	+	999	333	0	7835644 - 7836642	Nuclear
Brassica napus							
BnMAPKK1	JQ708028	+	1068	356	7	46341139-46343642	Nuclear
BnMAPKK2	JQ708029	-	1095	365	6	5711304-5713527	Extracellular
BnMAPKK3	JQ708030	-	1557	519	10	45241308-45245279	Extracellular
BnMAPKK4	JQ708031	+	1071	357	0	474756-476374	Nuclear
BnMAPKK5	KC246595	-	999	333	0	33278120-33279549	Nuclear
BnMAPKK6	JQ708032	-	1071	357	8	7673466-7678442	Nuclear
BnMAPKK8	XM_013841956	-	954	318	0	37811314-37812267	Nuclear
BnMAPKK9	JQ708033	-	921	307	0	16636545-16637662	Cytoplasmic
Brassica oleracea							
BoMAPKK1	Bol042272	+	1062	354	7	44087340-44089556	Nuclear
BoMAPKK2	Bol020940	+	1095	365	6	4755001-4756893	Extracellular
BoMAPKK4	Bol031584	-	1017	339	0	22698835-22699851	Nuclear
BoMAPKK5-1	Bol018796	-	984	328	0	29231424-29232407	Nuclear
BoMAPKK5-2	Bol026624	+	642	214	0	24055009-24055650	Nuclear
BoMAPKK5-3	Bol026625	+	609	203	0	24050558-24051166	Nuclear
BoMAPKK6-1	Bol026078	+	1077	359	7	6163978-6165928	Nuclear
BoMAPKK6-2	Bol009929	-	771	257	4	24750857-24752136	Extracellular
BoMAPKK7	Bol039425	-	1062	354	0	16720189-16721250	Cytoplasmic
BoMAPKK9	Bol040005	+	924	308	0	33353975-33354898	Cytoplasmic
Brassica rapa							
BrMAPKK1	BR03G48960	+	1068	356	7	26219071-26221288	Nuclear
BrMAPKK2	BR01G07590	+	1095	365	6	3717557-3719415	Extracellular
BrMAPKK3	BR04G09820	+	1557	519	8	7743699-7745965	Extracellular
BrMAPKK4-1	BR05G17730	-	1014	338	0	11149868-11150881	Nuclear
BrMAPKK4-2	BR08G02520	-	1068	356	0	1859660-1860727	Nuclear
BrMAPKK5-1	BR01G29490	-	996	332	0	20889624-20890619	Nuclear
BrMAPKK5-2	BR03G36620	+	1140	380	1	18630358-18633581	Nuclear
BrMAPKK5-3	BR03G36630	+	993	331	0	18638381-18639373	Nuclear
BrMAPKK6-1	BR03G12020	+	1047	349	6	5405408-5407159	Nuclear
BrMAPKK6-2	BR10G09810	-	1071	357	7	7477887-7479781	Nuclear
BrMAPKK8	BR02G34140	-	927	309	0	23201987-23202913	Nuclear
BrMAPKK9-1	BR02G22200	-	921	307	0	13124885-13125805	Cytoplasmic
BrMAPKK9-2	BR07G20830	+	927	309	0	15317633-15318559	Nuclear

BrMAPKK10	BR09G26620	-	927	309	0	20147438-20148364	Cytoplasmic
Capsella rubella							
CruMAPKK1-1	CRU_006G25040	+	1197	398	7	10285318-10287179	Cytoplasmic
CruMAPKK1-2	CRU_007G14010	-	1110	370	6	5561729-5565045	Nuclear
CruMAPKK2	CRU_004G14330	+	1092	364	7	8939787-8942115	Extracellular
CruMAPKK3	CRU_007G33860	-	1563	521	8	16055295-16057766	Extracellular
CruMAPKK4	CRU_001G38610	+	1107	369	0	17892574-17893680	Nuclear
CruMAPKK5	CRU_003G20230	+	1020	340	0	7638482-7639501	Nuclear
CruMAPKK6	CRU_008G16640	-	1074	358	7	9041811-9043758	Nuclear
CruMAPKK7	CRU_001G17210	+	936	312	0	6330821-6331756	Nuclear
CruMAPKK8	CRU_003G05430	+	954	318	0	1920830-1921783	Nuclear
CruMAPKK9	CRU_002G19080	-	939	313	0	11202114-11203052	Nuclear
CruMAPKK10	CRU_001G29220	+	942	314	0	11340851-11341792	Cytoplasmic
Capsicum annuum							
CaMAPKK2	CA00G74230	-	1074	358	7	5779660–5782151	Nuclear
CaMAPKK3	CA03G23860	+	1122	374	6	227498017–227503127	Cytoplasmic
CaMAPKK5	CA03G36820	+	1140	380	0	257411669–257412808	Nuclear
CaMAPKK6	CA00G86340	-	1116	372	7	10208073–10210986	Nuclear
CaMAPKK9	CA03G22790	-	984	328	0	224956092–224957075	Nuclear
Carica papaya							
CpMAPKK1	CP00371G00040	+	1065	355	7	32061-35628	Nuclear
CpMAPKK3	CP00108G00610	+	1557	519	8	645059-648769	Cytoplasmic
CpMAPKK5	CP46236G00010	+	651	217	0	38-688	Nuclear
CpMAPKK6	CP00078G00290	-	1065	355	7	413658-419291	Nuclear
CpMAPKK7	CP00003G03590	+	894	298	2	2447698-2448661	Nuclear
CpMAPKK9-1	CP00756G00010	+	441	147	1	73-545	Cytoplasmic
CpMAPKK9-2	CP01155G00030	-	909	303	0	7964-8872	Nuclear
CpMAPKK9-3	CP01155G00040	-	399	133	0	9314-9712	Extracellular
CpMAPKK10	CP00090G00140	-	432	144	0	356723-357154	Cytoplasmic
Chlamydomonas reinhardtii							
CrMAPKK2	CR13G00450	-	1422	474	6	251151-254201	Nuclear
CrMAPKK3	CR06G00090	-	1359	453	13	52657-56143	Mitochondrial
Citrullus lanatus							
ClMAPKK1	CL11G17340	-	1086	362	7	25376095-25378358	Cytoplasmic
ClMAPKK2	CL03G15020	+	1023	341	7	26070985-26074198	Cytoplasmic
ClMAPKK3	CL10G12060	-	1557	519	8	19766138-19772039	Nuclear
ClMAPKK4	CL07G12000	-	1110	370	0	24103885-24104994	Nuclear
ClMAPKK6	CL11G16940	-	1065	355	7	25016400-25019647	Cytoplasmic
ClMAPKK9	CL04G10340	+	636	212	1	22031703-22032406	Nuclear
Citrus sinensis							

CsMAPKK2	CS00112G00060	+	1176	392	9	194168-200414	Nuclear
CsMAPKK3	CS00116G00190	-	1557	519	8	188972-193529	Cytoplasmic
CsMAPKK4	CS00729G00050	+	1143	381	0	41219-42361	Nuclear
CsMAPKK6	CS00027G00740	-	1065	355	7	1130519-1134419	Nuclear
CsMAPKK8	CS06934G00010	-	900	300	1	1751-2712	Nuclear
CsMAPKK9	CS00315G00070	-	972	324	0	155512-156483	Mitochondrial
CsMAPKK10	CS00488G00010	+	1014	338	0	109992-111005	Mitochondrial
Coffea canephora							
CcMAPKK2	Cc06_g11750	-	1065	355	7	9510959-9514778	Cytoplasmic
CcMAPKK3	Cc00_g04720	+	1557	519	8	35910979-35918723	Cytoplasmic
CcMAPKK4	Cc04_g00500	+	1131	377	0	378966-380096	Nuclear
CcMAPKK6	Cc06_g10630	-	1716	572	12	8671731-8683688	Nuclear
CcMAPKK9	Cc04_g09350	+	981	327	0	7787585-7788565	Mitochondrial
CcMAPKK10	Cc08_g03210	+	1014	338	0	4007742-4008755	Cytoplasmic
Cucumis melo							
CmMAPKK1	CM00003G07700	-	918	306	5	8838179-8839975	Cytoplasmic
CmMAPKK2	CM00025G02930	-	1023	341	7	2320452-2323356	Cytoplasmic
CmMAPKK3	CM00098G00010	+	1557	519	8	2307-6898	Cytoplasmic
CmMAPKK5	CM00083G00040	-	1107	369	0	55987-57093	Nuclear
CmMAPKK6	CM00003G07280	-	1083	361	7	8572226-8575579	Mitochondrial
CmMAPKK9	CM00001G02470	-	966	322	0	1559662-1560627	Nuclear
Cucumis sativus							
CsaMAPKK2-1	Csa1M589750.1	-	1068	356	7	22388880–22391657	Cytoplasmic
CsaMAPKK2-2	Csa2M000340.1	+	1062	354	7	186835–189195	Cytoplasmic
CsaMAPKK3	Csa3M839800.1	+	1557	519	8	33665265–33669765	Cytoplasmic
CsaMAPKK4	Csa3M651720.1	-	1107	369	0	25634070–25635176	Nuclear
CsaMAPKK6	Csa2M000780.1	+	1077	359	7	443066–445832	Mitochondrial
CsaMAPKK9	Csa1M042980.1	-	963	321	0	4620708–4621670	Nuclear
Dianthus caryophyllus							
DcMAPKK1	Dca12122.1	+	1062	354	7	234145-238405	Cytoplasmic
DcMAPKK3	Dca49240.1	-	1527	509	8	64009-66956	Nuclear
DcMAPKK4	Dca4424.1	+	903	301	0	63849-65106	Nuclear
DcMAPKK5	Dca43553.1	+	1155	385	0	1514-2880	Nuclear
DcMAPKK6-1	Dca32756.1	+	1143	381	8	5780-9527	Extracellular
DcMAPKK6-2	Dca43468.1	+	777	259	3	7474-8960	Extracellular
DcMAPKK8	Dca52805.1	+	957	319	0	328871-330189	Mitochondrial
DcMAPKK10	Dca57202.1	+	972	324	1	62589-63705	Nuclear
Eucalyptus grandis							
EgMAPKK2	EG0003G06980	+	966	322	6	13769849-13772922	Extracellular
EgMAPKK3	EG0009G14680	-	1557	519	8	27417709-27422601	Cytoplasmic
EgMAPKK6	EG0003G28720	+	1077	359	7	70646700-70651230	Nuclear
EgMAPKK7	EG0011G19010	-	963	321	1	29993135-29994186	Nuclear
EgMAPKK9	EG0008G04760	-	996	332	0	7742233-7743228	Nuclear

EgMAPKK10	EG0005G02270	-	978	326	0	2486110-2487084	Cytoplasmic
Fragaria vesca							
FvMAPKK1	FV1G09520	-	1236	412	9	6713797-6716867	Nuclear
FvMAPKK8	FV5G02850	+	300	100	0	1456315-1456614	Nuclear
FvMAPKK9-1	FV5G16270	+	1218	406	1	10258248-10262858	Nuclear
FvMAPKK9-2	FV5G02870	+	1053	351	0	1459944-1460996	Nuclear
FvMAPKK9-3	FV5G02840	+	735	245	0	1451263-1451997	Nuclear
Glycine max							
GmMAPKK1-1	GM15G18860	-	1080	360	7	15770810-15775362	Cytoplasmic
GmMAPKK1-2	GM09G07661	-	807	269	8	6578141-6584984	Cytoplasmic
GmMAPKK2-1	GM13G16650	-	1071	357	7	20566417-20570315	Cytoplasmic
GmMAPKK2-2	GM17G06020	+	1071	357	7	4243961-4247916	Extracellular
GmMAPKK3-1	GM05G08720	+	1557	519	8	8603268-8607204	Cytoplasmic
GmMAPKK3-2	GM19G00220	+	1581	527	8	30059-34226	Cytoplasmic
GmMAPKK4	GM07G00520	-	1056	352	0	246220-247275	Nuclear
GmMAPKK5	GM08G23900	+	1095	365	0	18192648-18193742	Nuclear
GmMAPKK6-1	GM10G15850	+	1071	357	7	18504593-18521277	Nuclear
GmMAPKK6-2	GM02G32980	-	1065	355	7	36290448-36301474	Nuclear
GmMAPKK7	GM09G30300	+	960	320	0	37144106-37145065	Mitochondrial
GmMAPKK8	GM09G30310	+	759	253	1	37149613-37150510	Extracellular
GmMAPKK9	GM07G11910	-	957	319	0	10172403-10173359	Mitochondrial
GmMAPKK10	GM01G01980	-	1014	338	0	1537140-1538153	Extracellular
Gossypium raimondii							
GrMAPKK1	GR09G03380	-	1086	362	7	2527973-2530544	Cytoplasmic
GrMAPKK2-1	GR01G01360	-	1089	363	7	1295877-1299311	Extracellular
GrMAPKK2-2	GR10G08530	-	1095	365	4	12849778-12851815	Mitochondrial
GrMAPKK3	GR07G07560	-	1557	519	8	5329676-5332968	Nuclear
GrMAPKK4	GR10G22180	-	1050	350	0	59219186-59220235	Nuclear
GrMAPKK5	GR09G11780	+	1053	351	0	8712477-8713529	Nuclear
GrMAPKK6	GR13G22960	+	1065	355	7	54835660-54838817	Nuclear
GrMAPKK7	GR08G29170	-	972	324	0	56597189-56598160	Mitochondrial
GrMAPKK9	GR08G29180	-	966	322	0	56603652-56604617	Mitochondrial
GrMAPKK10-1	GR03G18400	+	963	322	0	45514142-45515104	Mitochondrial
GrMAPKK10-2	GR08G22510	-	963	321	0	51168182-51169144	Cytoplasmic
Hordeum vulgare							
HvMAPKK1	HV135797G00010	-	1047	349	8	2111-5402	Nuclear
HvMAPKK3	HV36969G00010	+	1125	375	8	999-7163	Cytoplasmic
HvMAPKK4	HV160058G00010	-	1041	347	0	2330-3040	Nuclear
HvMAPKK6	HV135110G00010	-	1068	356	5	141-4125	Cytoplasmic
HvMAPKK10-1	HV42885G00010	+	363	121	0	41-834	Mitochondrial
HvMAPKK10-2	HV7730G00010	-	996	332	0	6666-7625	Nuclear
Jatropha curcas							
JcMAPKK1	XP_012066794	-	1968	656	10	999261-1004850	Nuclear

JcMAPKK2-1	Jcr4S00005.240	-	2007	669	8	2718840-2723412	Nuclear
JcMAPKK2-2	Jcr4S07011.20	-	1383	461	9	2862983-2867838	Nuclear
JcMAPKK3	Jcr4S00518.50	+	1440	480	10	570791-575688	Cytoplasmic
JcMAPKK5-1	Jcr4S10674.40	+	1032	344	0	119681-121290	Nuclear
JcMAPKK5-2	Jcr4S26937.20	+	1059	353	0	119681-121290	Nuclear
JcMAPKK6	Jcr4S01567.130	-	1065	355	7	2142318-2145827	Nuclear
JcMAPKK7	Jcr4S00008.310	+	984	328	0	2340359-2341342	Extracellular
JcMAPKK9	Jcr4S08175.20	-	888	296	0	39530-40755	Nuclear
JcMAPKK10	Jcr4S12790.10	+	1050	350	0	263800-264768	Mitochondrial
Lotus japonicus							
LjMAPKK1	chr6.CM0013.1740.r2.d	+	927	309	7	17116393-17119281	Extracellular
LjMAPKK2	chr4.CM0297.320.r2.m	-	1074	358	7	31300747-31304728	Nuclear
LjMAPKK3	chr2.CM0263.270.r2.m	+	1581	527	8	15153219-15157589	Cytoplasmic
LjMAPKK4	chr3.LjB21L17.150.r2.a	-	1122	374	0	28798445-28799563	Nuclear
LjMAPKK6	chr2.CM0065.640.r2.d	+	972	324	6	13536732-13540679	Nuclear
LjMAPKK9	LjSGA_014749.1.1	-	1065	355	0	470-1538	Extracellular
LjMAPKK10	LjSGA_023606.1	+	1008	336	0	771-1778	Extracellular
Malus domestica							
MdMAPKK2	MD02G007060	-	966	322	5	6497216-6502072	Nuclear
MdMAPKK3	MD09G001900	-	1665	555	7	1605591-1608205	Cytoplasmic
MdMAPKK4-1	MD09G012300	-	1071	357	0	12276753-12277823	Nuclear
MdMAPKK4-2	MD17G012670	-	1074	358	0	12391994-12393067	Nuclear
MdMAPKK6-1	MD02G005910	-	1236	412	8	5468518-5471660	Nuclear
MdMAPKK6-2	MD15G014130	-	1083	361	7	14039352-14042564	Cytoplasmic
MdMAPKK9-1	MD00G332250	-	945	315	0	12121-13065	Mitochondrial
MdMAPKK9-2	MD16G011070	-	951	317	0	10247301-10248251	Nuclear
MdMAPKK9-3	MD06G019470	-	969	323	0	24033774-24034742	Mitochondrial
Manihot esculenta							
MeMAPKK1-1	ME09809G00210	+	1095	365	7	552716-554876	Nuclear
MeMAPKK1-2	ME09809G00230	+	1104	368	7	560841-564048	Nuclear
MeMAPKK2-1	ME07318G00190	+	765	255	5	179969-182357	Nuclear
MeMAPKK2-2	ME10493G00760	+	1071	357	8	890642-896041	Nuclear
MeMAPKK3	ME05694G00070	+	1557	519	8	134421-138498	Cytoplasmic
MeMAPKK4	ME01027G00160	-	1071	357	0	479994-481064	Nuclear
MeMAPKK5	ME09974G00010	-	1074	358	0	15098-16171	Nuclear
MeMAPKK6	ME09501G00340	-	1008	336	7	347345-351265	Nuclear
MeMAPKK7	ME10870G00010	+	945	315	0	2647-3591	Extracellular
MeMAPKK9	ME11170G00070	+	966	322	0	108233-109198	Nuclear
MeMAPKK10	ME04795G00070	-	978	326	0	229273-230250	Mitochondrial
Medicago truncatula							
MtMAPKK1	MT2G040510	-	1083	361	7	17774667-17778046	Extracellular
MtMAPKK2	MT4G125800	+	1140	380	8	52212057-52216489	Nuclear
MtMAPKK3	MT6G005210	-	1560	520	8	650766-656396	Cytoplasmic

MtMAPKK4	MT4G005830	-	1101	367	0	382504-383604	Nuclear
MtMAPKK6	MT6G090470	-	1065	355	7	34053017-34058389	Nuclear
MtMAPKK9	MT6G071280	-	975	325	0	26348430-26349404	Mitochondrial
Micromonas pusilla							
MpMAPKK3	MP14G01300	+	1395	465	5	340319 - 343127	Extracellular
MpMAPKK6	MP06G05440	-	984	328	0	1079440 - 1080426	Cytoplasmic
Musa acuminata							
MaMAPKK1	MA10G21630	-	1272	424	8	26898285-26901045	Nuclear
MaMAPKK2-1	MA07G09900	+	1053	351	7	7853024-7858432	Extracellular
MaMAPKK2-2	MA10G28080	-	1050	350	8	30628842-30633612	Cytoplasmic
MaMAPKK3	MA09G19160	-	891	297	4	14180466-14187281	Cytoplasmic
MaMAPKK4	MA03G05070	+	1365	455	2	3285271-3288887	Mitochondrial
MaMAPKK5	MA04G06010	-	999	333	1	4547699-4548791	Nuclear
MaMAPKK6	MA09G00100	+	936	312	6	154641-168166	Cytoplasmic
MaMAPKK10-1	MA11G09660	+	867	289	1	7389215-7390276	Nuclear
MaMAPKK10-2	MA06G11530	-	891	297	2	7491564-7492810	Mitochondrial
Oryza sativa							
OsMAPKK1	Os06G05520	-	1059	353	8	2499218-2502776	Nuclear
OsMAPKK3	Os06G27890	-	1572	524	8	15811653-15821588	Cytoplasmic
OsMAPKK4	Os02G54600	-	1110	370	0	33442732-33443841	Nuclear
OsMAPKK5	Os06G09180	+	1029	343	0	4616701-4617729	Nuclear
OsMAPKK6	Os01G32660	+	1068	356	7	17905076-17909767	Cytoplasmic
OsMAPKK10-1	Os02G46760	+	1023	341	0	28552739-28553761	Mitochondrial
OsMAPKK10-2	Os03G12390	-	1020	340	0	6545395-6546414	Nuclear
OsMAPKK10-3	Os03G50550	+	1038	346	0	28861857-28862894	Nuclear
Ostreococcus lucimarinus							
OlMAPKK6	OL04G03760	+	801	267	0	677099-677899	Cytoplasmic
Phaeodactylum tricornutum							
PtiMAPKK1	PTI_01G05340	+	489	163	0	1317484 - 1317972	Cytoplasmic
Phaseolus vulgaris							
PvMAPKK1	Phvul.009G229800.1	+	1071	357	7	33934787-33939650	Cytoplasmic
PvMAPKK2	Phvul.003G134100.1	+	1071	357	7	32499664-32504578	Cytoplasmic
PvMAPKK3-1	Phvul.004G010400.1	+	1560	520	8	1015240-1019248	Cytoplasmic
PvMAPKK3-2	Phvul.007G112700.1	+	762	254	5	14665196-14667460	Nuclear
PvMAPKK4	Phvul.010G163000.1	+	1092	364	0	43013417-43015249	Nuclear
PvMAPKK6	Phvul.004G174500.1	+	1065	355	7	45487276-45491481	Nuclear
PvMAPKK8	Phvul.004G147900.1	-	969	323	1	42842574-42844194	Extracellular
PvMAPKK9	Phvul.004G147800.1	+	954	318	0	42835942-42837073	Mitochondrial
PvMAPKK10	Phvul.002G138400.1	-	1026	342	0	27119115-27120140	Extracellular
Physcomitrella patens							
PpMAPKK3-1	PP00092G00080	-	1560	520	9	293057-298328	Cytoplasmic
PpMAPKK3-2	PP00151G00010	+	1602	534	9	38932-45102	Cytoplasmic
PpMAPKK6-1	PP00050G00300	-	1041	347	7	837586-840389	Nuclear

PpMAPKK6-2	PP00106G00380	+	1029	343	7	495895-498624	Mitochondrial
PpMAPKK6-3	PP00032G01020	+	1059	353	7	1521818-1524830	Extracellular
PpMAPKK7	PP00114G00500	+	1107	369	0	495451-496557	Mitochondrial
PpMAPKK9	PP00016G01540	-	1038	346	0	2473459-2474496	Extracellular
Picea abies							
PaMAPKK3	MA_41206g0010	+	402	134	2	15231-22535	Nuclear
PaMAPKK4-1	MA_10192951g0010	-	702	234	1	528-2598	Nuclear
PaMAPKK4-2	MA_10192951g0020	+	567	189	0	2616-3222	Mitochondrial
PaMAPKK6-1	MA_177636g0010	-	924	308	4	15320-21032	Extracellular
PaMAPKK6-2	MA_206154g0010	-	687	229	3	1385-4959	Extracellular
PaMAPKK7	MA_10372232g0010	-	666	222	1	3878-4645	Nuclear
PaMAPKK9	MA_10194g0020	+	1026	342	0	13091-14156	Nuclear
Populus trichocarpa							
PtMAPKK2-1	PT18G05080	+	1143	381	8	5253125-5256398	Extracellular
PtMAPKK2-2	PT06G14650	+	1068	356	7	12509909-12512808	Nuclear
PtMAPKK3	PT01G34550	-	1566	522	8	35096866-35101233	Cytoplasmic
PtMAPKK4	PT10G24930	-	1059	353	0	22227003-22228061	Nuclear
PtMAPKK5	PT08G00980	+	1098	366	1	541082-543959	Nuclear
PtMAPKK6	PT18G06850	+	1074	358	7	8739286-8741486	Nuclear
PtMAPKK7	PT15G03070	-	966	322	0	2468208-2469173	Nuclear
PtMAPKK8-1	PT08G18370	+	963	321	0	12576300-12577262	Cytoplasmic
PtMAPKK8-2	PT10G04950	-	861	287	0	7999010-7999870	Cytoplasmic
PtMAPKK9	PT12G04320	+	963	321	0	3906908-3907870	Nuclear
PtMAPKK10	PT01G13880	-	999	333	0	11150054-11151052	Mitochondrial
Prunus persica							
PpeMAPKK2	PPE_007G22450	+	1065	355	7	19344356-19347068	Extracellular
PpeMAPKK3	PPE_003G26810	+	1557	519	8	20748037-20750824	Nuclear
PpeMAPKK5	PPE_003G15010	-	1062	354	0	13377712-13378773	Nuclear
PpeMAPKK6	PPE_007G23740	+	1071	357	7	20030323-20032984	Cytoplasmic
PpeMAPKK7	PPE_001G05740	-	978	326	0	4140857-4141834	Nuclear
PpeMAPKK8	PPE_005G23760	-	675	225	0	17916853-17917527	Extracellular
PpeMAPKK9-1	PPE_001G08120	+	966	322	0	6177732-6178697	Nuclear
PpeMAPKK9-2	PPE_001G18890	+	978	326	0	16796844-16797821	Nuclear
PpeMAPKK9-3	PPE_001G07660	+	966	322	0	5694287-5695252	Extracellular
PpeMAPKK10	PPE_005G03470	-	1035	345	0	3539881-3540915	Extracellular
Ricinus communis							
RcMAPKK2	RC29912G00340	+	1023	341	8	208493-212275	Extracellular
RcMAPKK3	RC29917G00620	+	1557	519	8	412947-416627	Cytoplasmic
RcMAPKK4	RC29748G00070	+	1158	386	0	178249-179406	Nuclear
RcMAPKK6	RC29912G01450	+	1068	356	7	916097-918839	Nuclear
RcMAPKK9	RC29929G02980	+	795	265	2	1715143-1716875	Nuclear
RcMAPKK10	RC30054G00130	-	909	303	1	89225-90216	Cytoplasmic
Saccharina japonica							

SjMAPKK1	SJ16021	+	3651	1217	5	352089-358742	Extracellular
SjMAPKK3	SJ02374	+	951	317	6	84188-90880	Mitochondrial
Selaginella moellendorffii							
SmMAPKK3	SM00000G08420	+	1530	510	8	4566052-4568212	Nuclear
SmMAPKK7	SM00011G01810	+	1002	334	0	994152 - 995153	Mitochondrial
SmMAPKK9	SM00017G02550	-	990	330	1	1540728 - 1544918	Nuclear
Setaria italica							
SiMAPKK1	SI004G03650	-	1053	351	8	2444101-2447094	Nuclear
SiMAPKK3-1	SI006G16020	-	1395	465	8	28355000-28360476	Cytoplasmic
SiMAPKK3-2	SI001G31910	-	1572	524	8	37055238-37064012	Nuclear
SiMAPKK3-3	SI001G31920	-	1572	524	8	37074331-37082852	Nuclear
SiMAPKK4-1	SI001G36010	-	1101	367	0	40030826-40031926	Nuclear
SiMAPKK4-2	SI005G23750	+	1026	342	0	29300337-29301362	Nuclear
SiMAPKK5	SI004G04440	-	1035	345	0	3195480-3196514	Nuclear
SiMAPKK6-1	SI003G06250	-	1068	356	7	3943434-3947087	Cytoplasmic
SiMAPKK6-2	SI005G30780	-	1068	356	7	35560620-35564552	Cytoplasmic
SiMAPKK10-1	SI009G11680	-	993	331	1	7285595-7286599	Nuclear
SiMAPKK10-2	SI009G50030	+	996	332	0	52473836-52474831	Nuclear
SiMAPKK10-3	SI009G11700	-	999	333	0	7294995-7295993	Nuclear
Solanum lycopersicum							
SlMAPKK1	SL12G009020	–	1074	358	7	2321616–2325502	Cytoplasmic
SlMAPKK3	SL03G019850	+	1548	516	8	6757647–6762359	Cytoplasmic
SlMAPKK4	SL03G123800	–	1080	360	0	64579230–64580309	Nuclear
SlMAPKK6	SL03G119490	+	1065	355	7	62138086–62141226	Nuclear
SlMAPKK9	SL03G097920	+	1008	336	0	53756873–53757880	Mitochondrial
Solanum melongena							
SmeMAPKK2	Sme2.5_01917.1_g00010.1	+	1206	402	6	59396-63278	Extracellular
SmeMAPKK3	Sme2.5_00861.1_g00010.1	+	1668	556	10	62496-70032	Cytoplasmic
SmeMAPKK4	Sme2.5_09230.1_g00001.1	+	1113	371	0	1147-2259	Nuclear
SmeMAPKK9	Sme2.5_00088.1_g00017.1	-	978	326	0	175687-176664	Nuclear
Solanum tuberosum							
StMAPKK2	ST12G010200	-	1074	358	7	4598674-4603061	Cytoplasmic
StMAPKK3	ST03G024510	+	1548	516	8	37346637-37351154	Cytoplasmic
StMAPKK4	ST03G034170	+	1113	371	0	46695423-46696535	Nuclear
StMAPKK6	ST03G033030	+	1065	355	7	45592889-45596023	Nuclear
StMAPKK9	ST03G022560	+	1002	334	0	35395116-35396117	Mitochondrial
Sorghum bicolor							
SbMAPKK4	SB04g035370	-	1167	389	0	65186458-65187624	Nuclear
SbMAPKK5	SB10G006080	+	627	209	0	5491014-5491640	Cytoplasmic

SbMAPKK6	SB03g033190	-	1068	356	7	61560158-61564738	Cytoplasmic
SbMAPKK10-1	SB04g031130	-	1083	361	0	61121643-61122725	Mitochondrial
SbMAPKK10-2	SB01g042350	+	1014	338	0	65617716-65618729	Nuclear
SbMAPKK10-3	SB01G010180	-	981	327	0	9051911-9052891	Nuclear
SbMAPKK10-4	SB01g010200	-	1011	337	0	9066624-9067634	Nuclear
Thellungiella parvula							
TpMAPKK2	TP7G27620	-	1092	364	7	712523-714558	Extracellular
TpMAPKK3	TP7G01490	-	1560	520	9	584254-587341	Extracellular
TpMAPKK4	TP1G38310	+	1113	371	0	3805764-3806876	Nuclear
TpMAPKK5	TP3G19220	+	1080	360	0	6865234-6866313	Nuclear
TpMAPKK6	TP6G18360	-	1068	356	7	2681427-2683522	Nuclear
TpMAPKK8	TP4G05470	-	993	331	0	1892203-1893195	Cytoplasmic
TpMAPKK9-1	TP5G28400	-	933	311	0	2910331-2911263	Nuclear
TpMAPKK9-2	TP5G28670	-	933	311	0	34837-35769	Nuclear
TpMAPKK10	TP1G28580	-	930	310	0	11049665-11050594	Cytoplasmic
Theobroma cacao							
TcMAPKK1	TC0009G07910	+	1086	362	7	4321054-4323745	Extracellular
TcMAPKK2	TC0009G07920	+	3894	1298	9	4328179-4334097	Cytoplasmic
TcMAPKK3	TC0004G01620	+	1557	519	8	1062281-1065525	Cytoplasmic
TcMAPKK4	TC0006G01710	+	1092	364	0	1429053-1430144	Nuclear
TcMAPKK6	TC0009G09420	+	1065	355	7	5166972-5170641	Nuclear
TcMAPKK7	TC0002G26500	-	957	319	0	34542820-34543776	Extracellular
TcMAPKK9	TC0003G06310	-	963	321	0	8262111-8263073	Nuclear
TcMAPKK10	TC0003G30210	+	1005	335	0	33452043-33453047	Mitochondrial
Vitis vinifera							
VvMAPKK2	VV11G03170	+	1065	355	7	1417439–1424337	Extracellular
VvMAPKK3	VV14G11130	-	1557	519	8	27139003–27145873	Cytoplasmic
VvMAPKK5	VV09G01210	+	1188	396	4	19257788–19265261	Nuclear
VvMAPKK6	VV11G04310	+	1065	355	7	2377698–2381398	Nuclear
VvMAPKK9	VV17G07540	+	675	225	2	1537423–1538551	Mitochondrial
Volvox carteri							
VcMAPKK6	VC00006G02490	-	1248	416	7	2135412 - 2139078	Cytoplasmic
Zea mays							
ZmMAPKK1	ZM09G04800	+	621	207	5	19610168-19611586	Nuclear
ZmMAPKK3-1	ZM03G15310	+	921	307	4	111057835-111072907	Cytoplasmic
ZmMAPKK3-2	ZM05G06160	-	645	215	4	12697432-12707265	Nuclear
ZmMAPKK4	ZM05G42740	-	1344	358	0	212654565-212655188	Nuclear
ZmMAPKK5	ZM09G02500	+	1044	348	0	11928290-11929333	Nuclear
ZmMAPKK6	ZM03G33010	+	1068	356	7	201896107-201901195	Cytoplasmic
ZmMAPKK10-1	ZM05G37270	+	1128	376	0	199138547-199139674	Mitochondrial

ZmMAPKK10-2	ZM01G08460	-	1206	402	0	28609597-28610802	Nuclear
Homo sapiens							
HsMAPKK1	uc010bhq.4	-	1182	393	8	chr15:66797173..66901898	Cytoplasmic
HsMAPKK2	uc002lzk.4		1203	400	10	chr19:4090321-4124129	Cytoplasmic
HsMAPKK3	uc002gys.4	+	1044	347	11	chr17:21284921-21314230	Cytoplasmic
HsMAPKK4	uc002gnj.5	+	1200	399	11	chr17:12020887-12141260	Cytoplasmic
HsMAPKK5	uc002aqu.4	+	1347	448	14	chr15:67543336-67806750	Cytoplasmic
HsMAPKK6	uc002jij.4	+	1005	334	8	chr17:69414985-69541753	Cytoplasmic
HsMAPKK7	uc002mit.4	+	1260	419	6	chr19:7903945-7912431	Cytoplasmic
Saccharomyces cerevisiae							
ScPBS2	YJL128C	-	2007	668	0	Chr X:180103-178097	OuterMembrane
ScMAPKK1	YOR231W	+	1527	508	0	Chr XV:772601-774127	OuterMembrane
ScMAPKK2	YPL140C	-	1521	506	0	Chr XVI:289034-287514	OuterMembrane
ScSTE7	YDL159W	+	1548	515	0	Chr IV:172481-174028	OuterMembrane
Schizosaccharomyces pombe							
SpWis1	SPBC409.07c	-	1818	605	0	Chr II:1146633-1143731	OuterMembrane
SpByr1	SPAC1D4.13	+	1023	340	0	Chr 1: 667815-669742	Cytoplasmic
SpPek1	SPBC543.07	+	1092	363	0	Chr 2: 4312422-4314140	Cytoplasmic
