# Supplementary figures and images for: Comparative analysis of plant MKK gene family reveals novel expansion mechanism of the members and sheds new light on functional conservation
Source: BMC Genomics. 2018 May 29;19:407. doi: 10.1186/s12864-018-4793-8 (PMC5975520; doi:10.1186/s12864-018-4793-8)

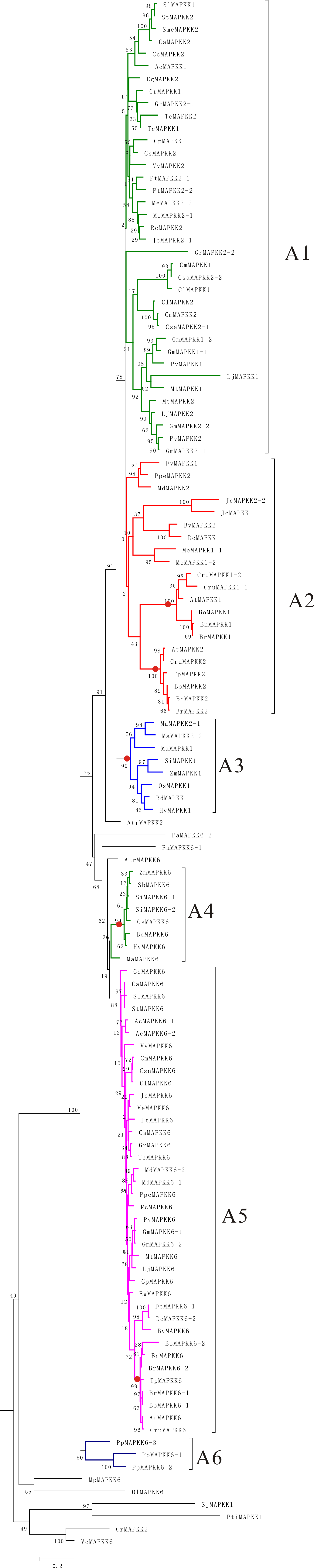

Supplement: Supplementary file 2 — Table S2. OrthoMCL automatic analysis about plant MAPKK. (TIF 40445 kb) [file 12864_2018_4793_MOESM2_ESM.tif]

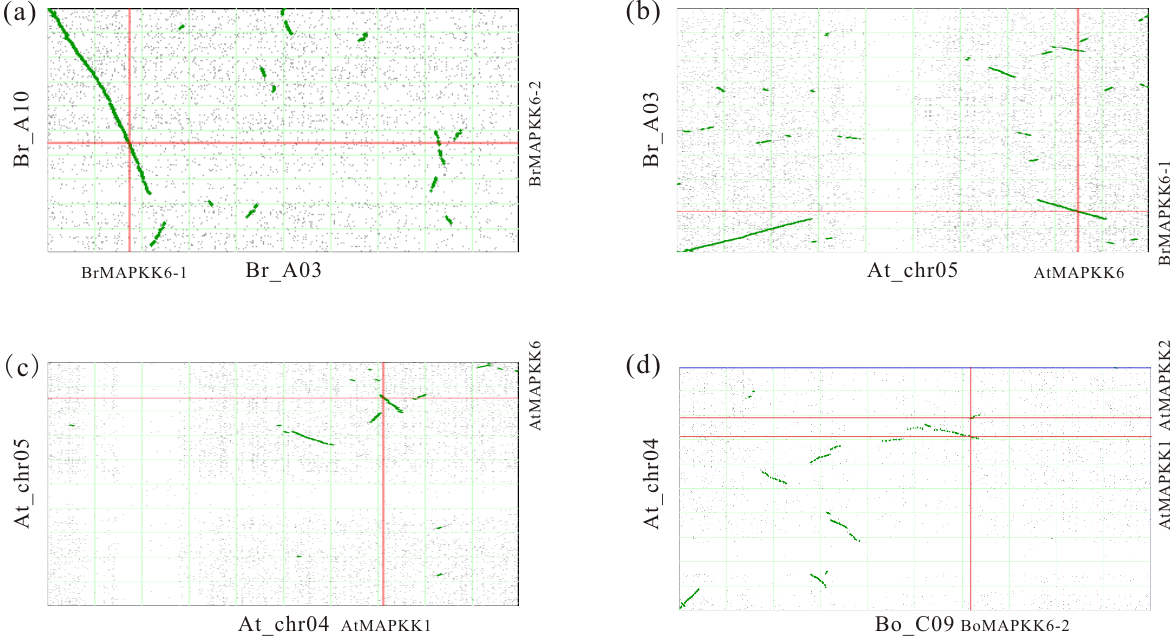

Supplement: Supplementary file 3 — Fig. S1. The exon/intron structures of plant MAPKK genes. (TIF 2182 kb) [file 12864_2018_4793_MOESM3_ESM.tif]

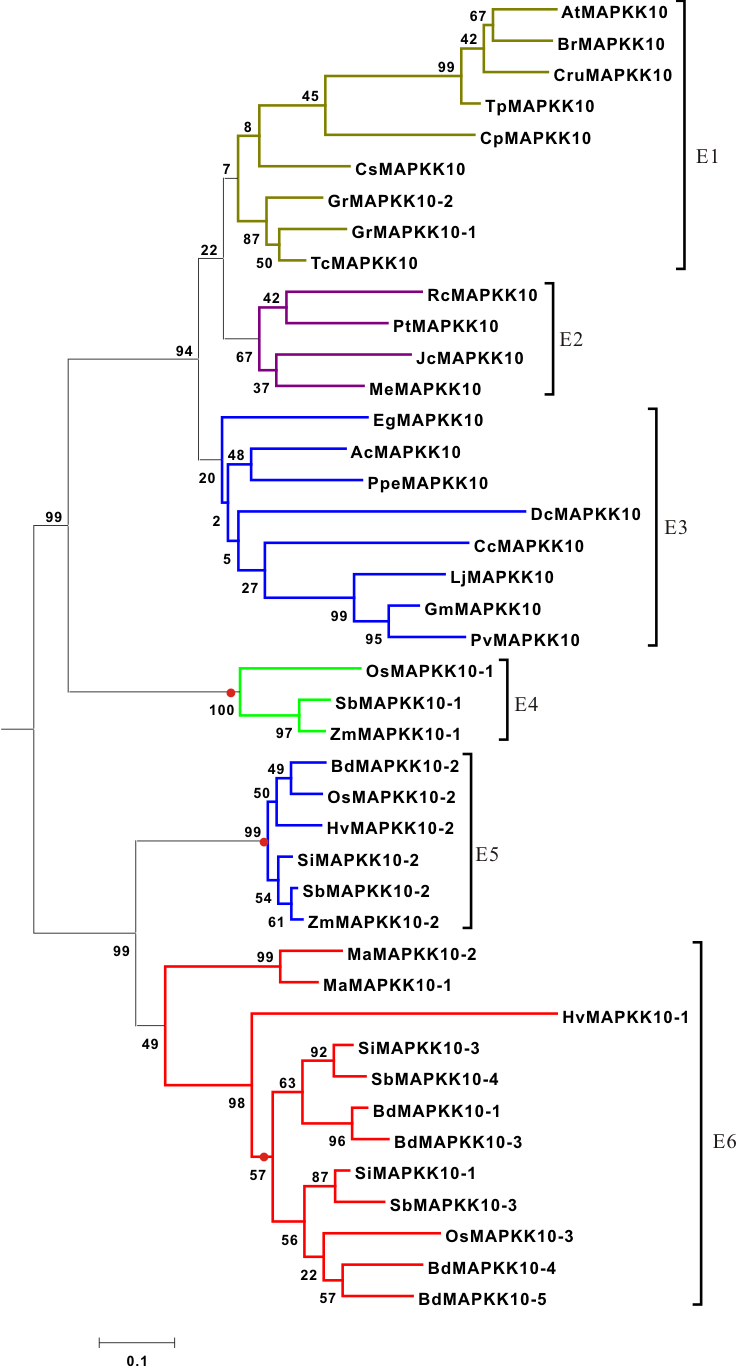

Supplement: Supplementary file 5 — Table S4. Table shows average amino acid composition of plant MAPKKs. (TIF 2947 kb) [file 12864_2018_4793_MOESM5_ESM.tif]

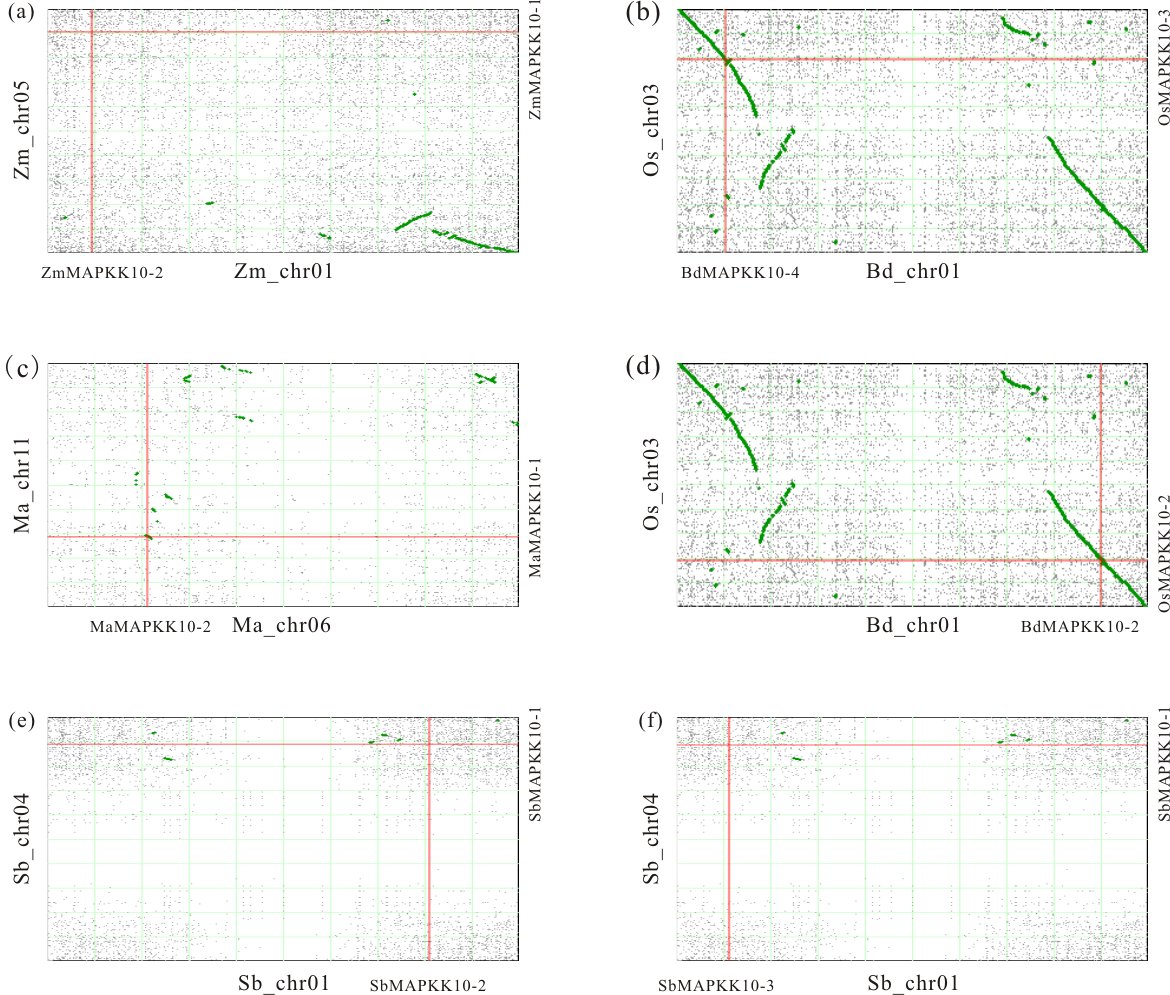

Supplement: Supplementary file 6 — Fig. S2. Weblogos represent the Nucleotide binding domain and the ATP binding site of each group. The stars indicate residues of functional or structural importance. (TIF 3413 kb) [file 12864_2018_4793_MOESM6_ESM.tif]

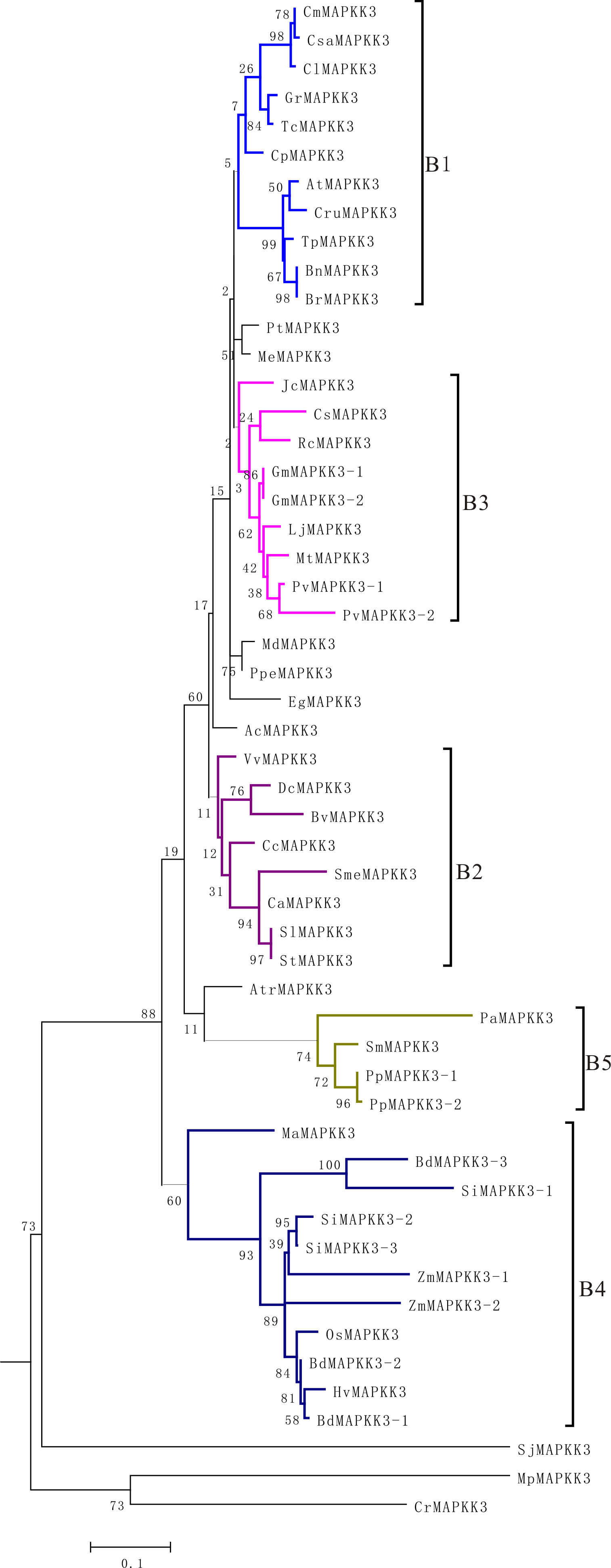

Supplement: Supplementary file 7 — Fig. S3. Weblogos represent the docking site (D-site) of each group. The stars indicate residues of functional or structural importance. (TIF 17233 kb) [file 12864_2018_4793_MOESM7_ESM.tif]

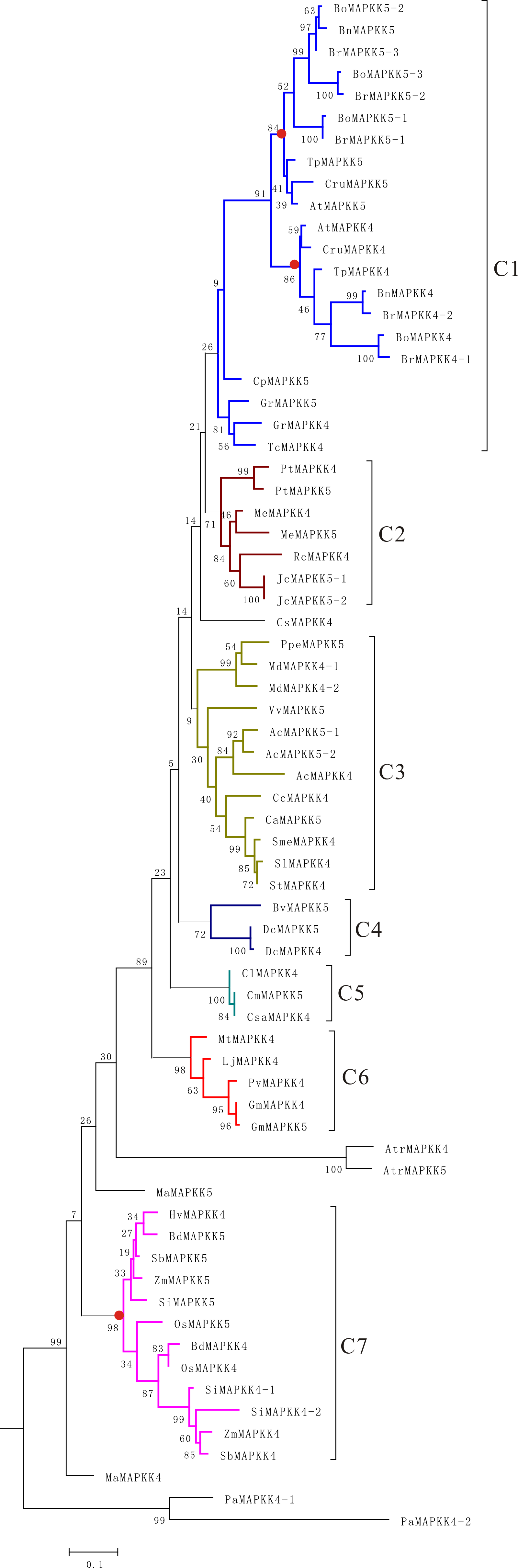

Supplement: Supplementary file 8 — Table S5. Molecular evolutionary analysis of the MAPKK genes in different motif. (TIF 22709 kb) [file 12864_2018_4793_MOESM8_ESM.tif]

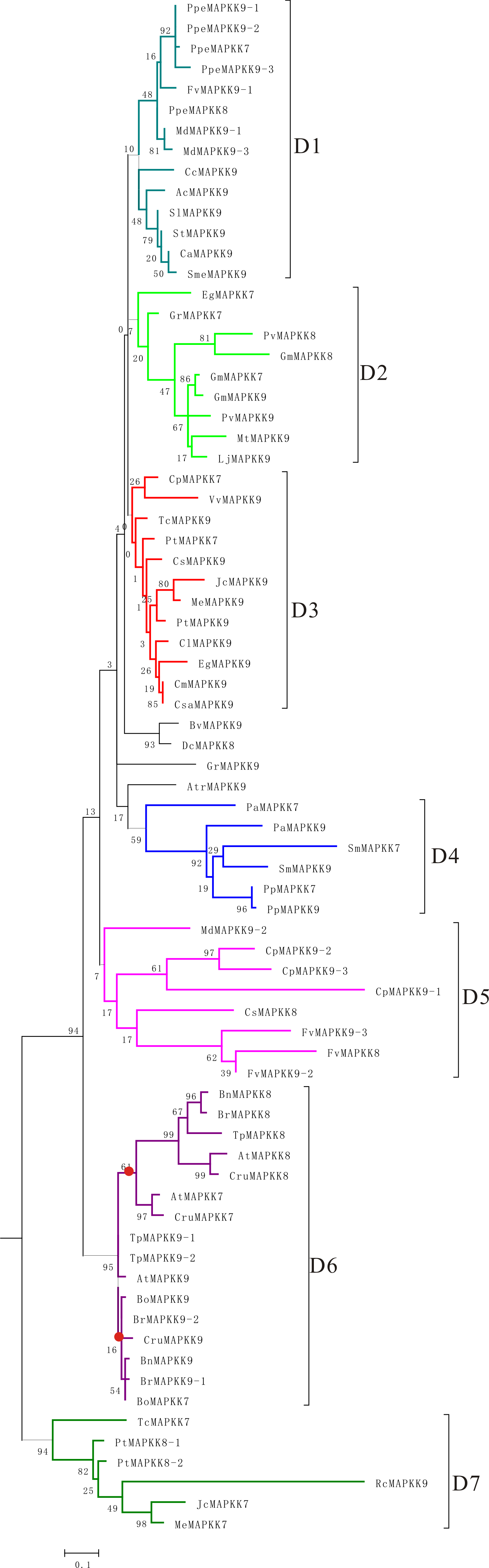

Supplement: Supplementary file 9 — Table S6. The duplicated gene pairs in the 51 plant genomes. (TIF 21703 kb) [file 12864_2018_4793_MOESM9_ESM.tif]

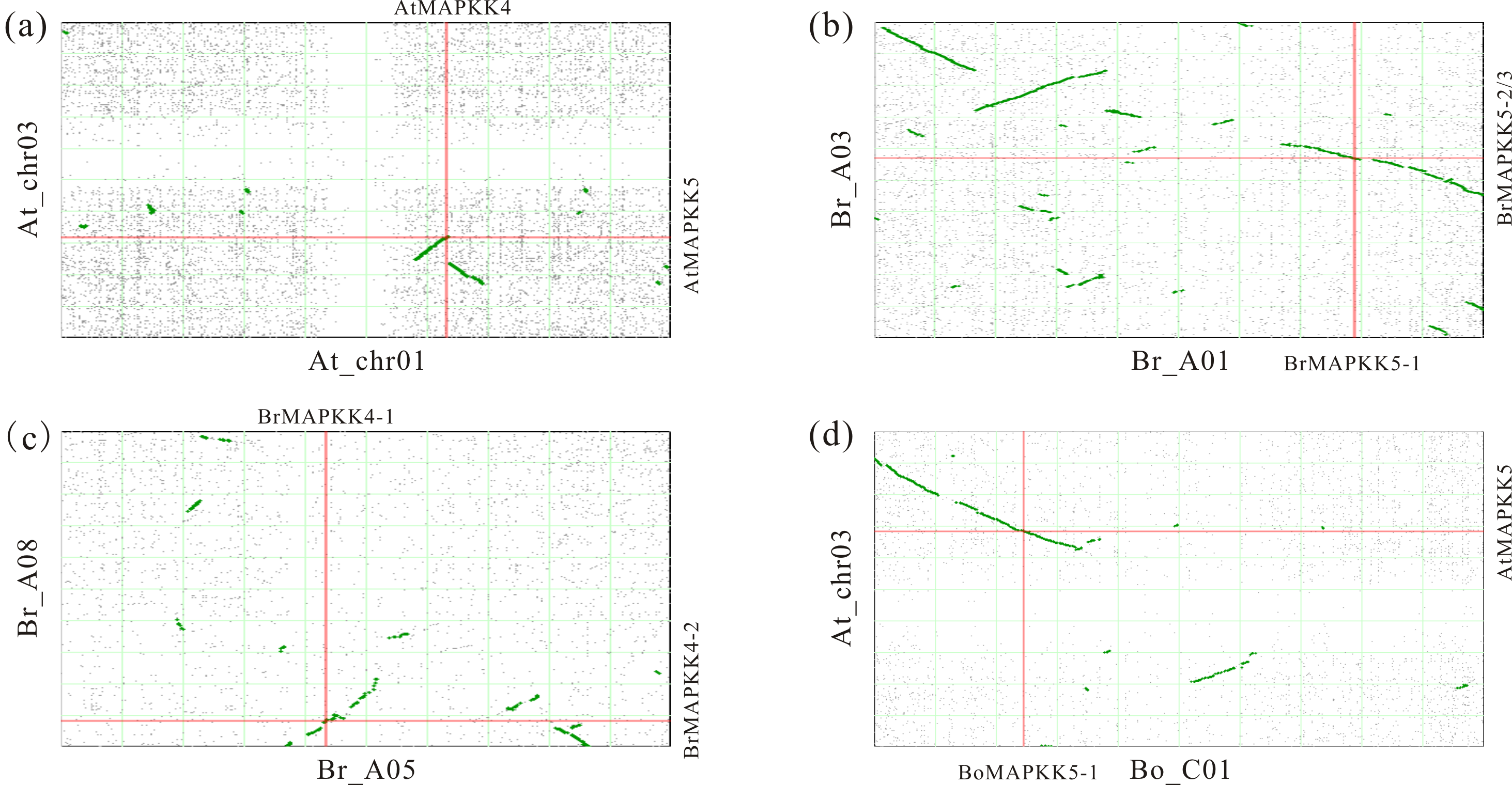

Supplement: Supplementary file 10 — Fig. S4. Maximum Likelihood phylogenetic trees of plant group A MAPKKs. The red circle represents duplication events. (TIF 13036 kb) [file 12864_2018_4793_MOESM10_ESM.tif]

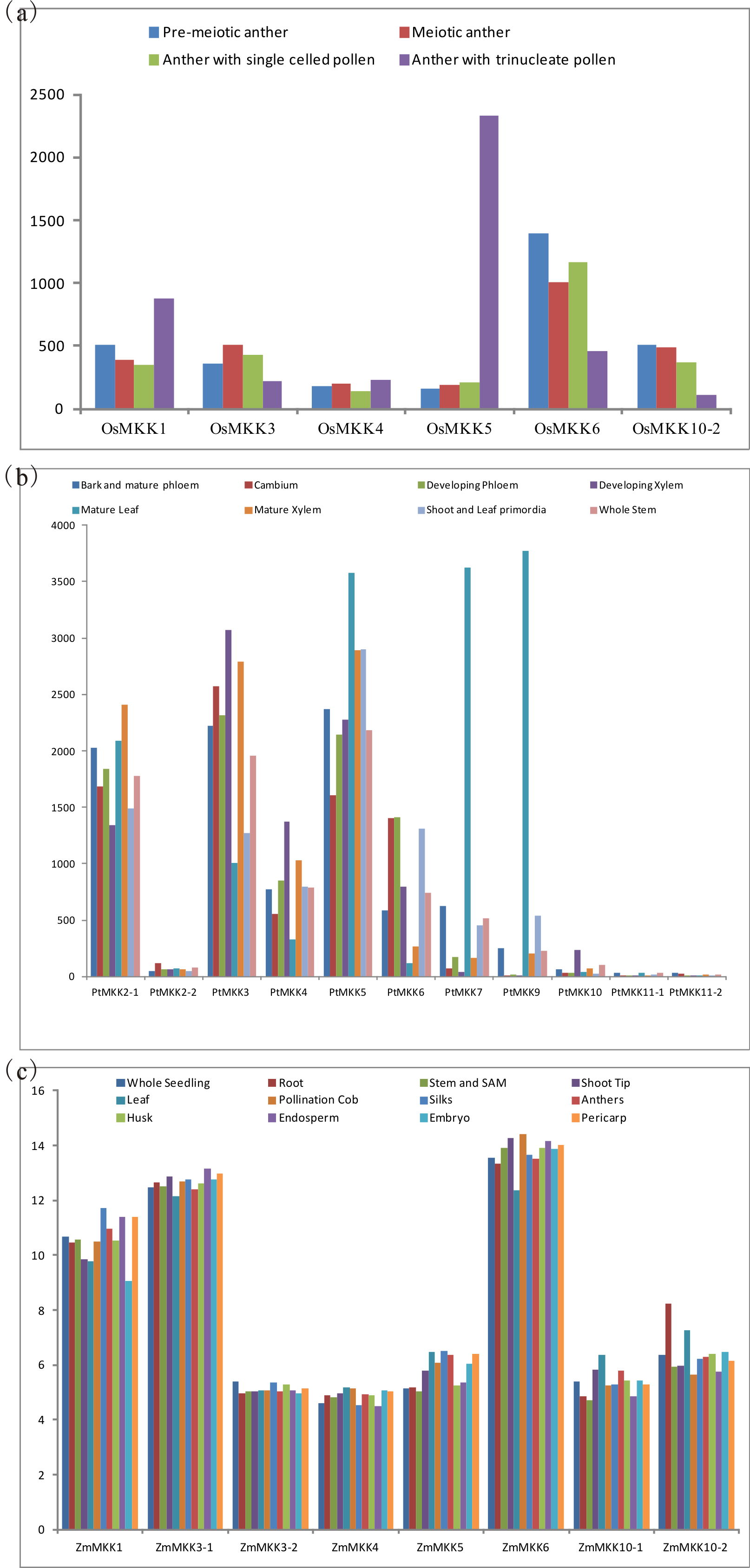

Supplement: Supplementary file 11 — Fig. S5. Syntenic proofs of Group A MAPKKs in Brassicaceae . (TIF 15972 kb) [file 12864_2018_4793_MOESM11_ESM.tif]

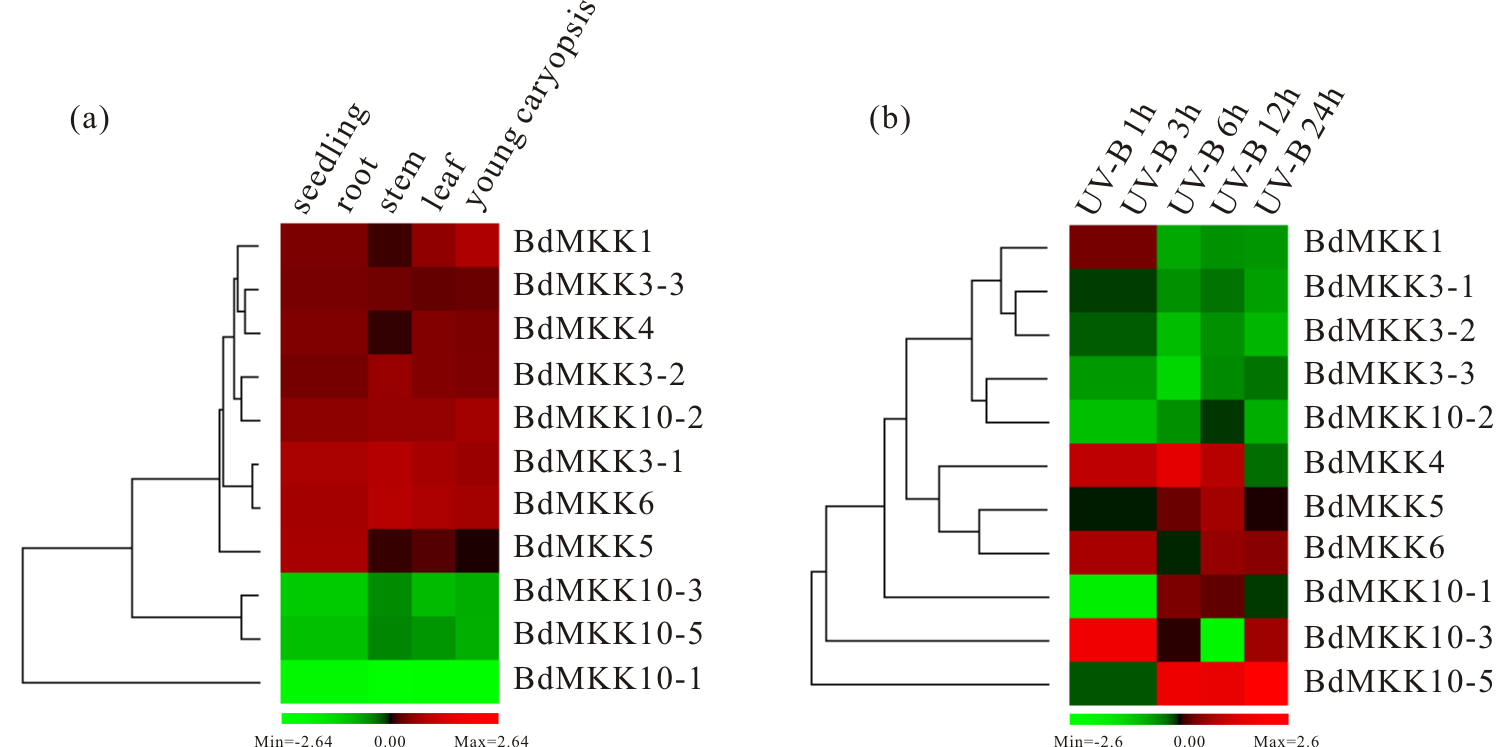

Supplement: Supplementary file 13 — Fig. S6. Maximum Likelihood phylogenetic trees of plant group E MAPKKs. The red circle represents duplication events. (TIF 3291 kb) [file 12864_2018_4793_MOESM13_ESM.tif]

**Fig.S1** The exon/intron structure of plant MAPKKs genes.

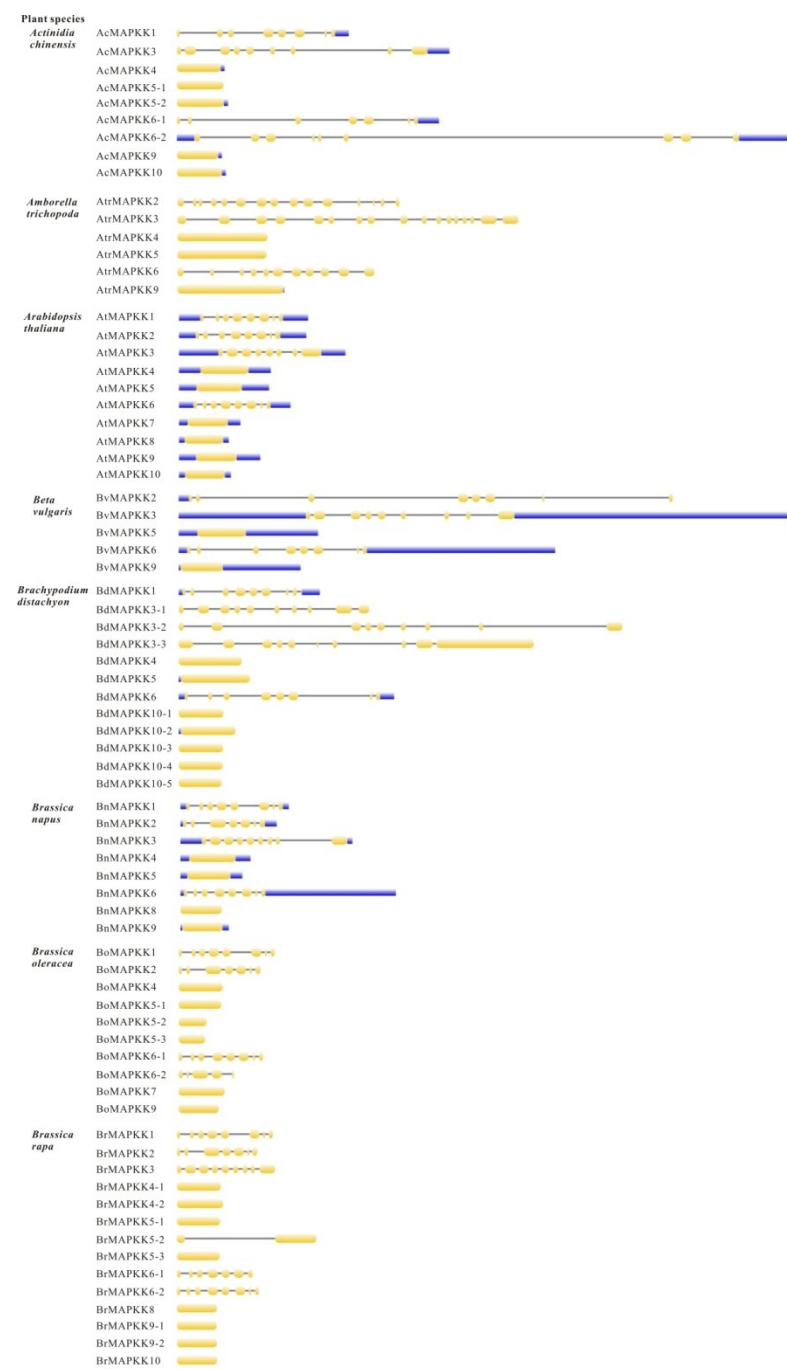

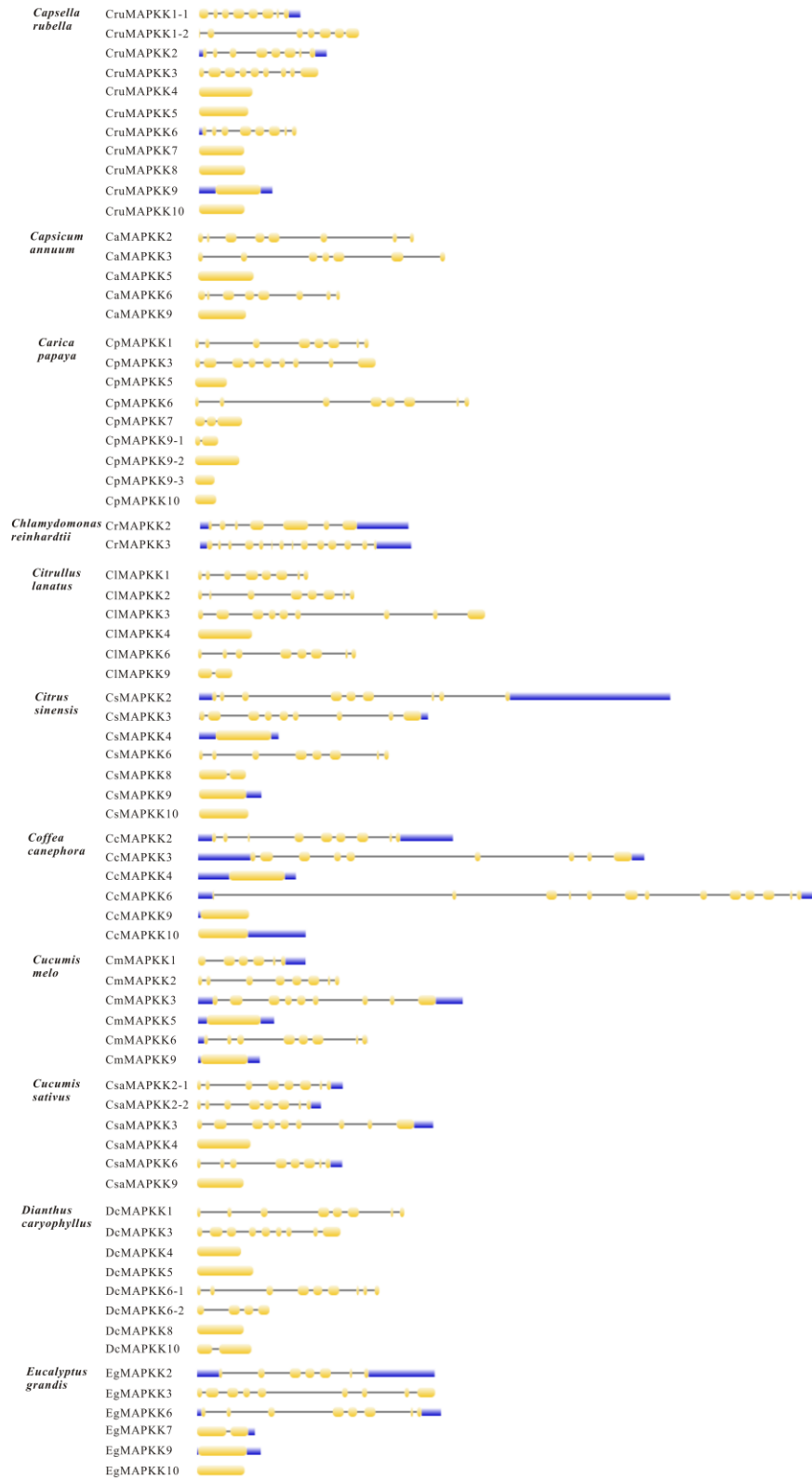

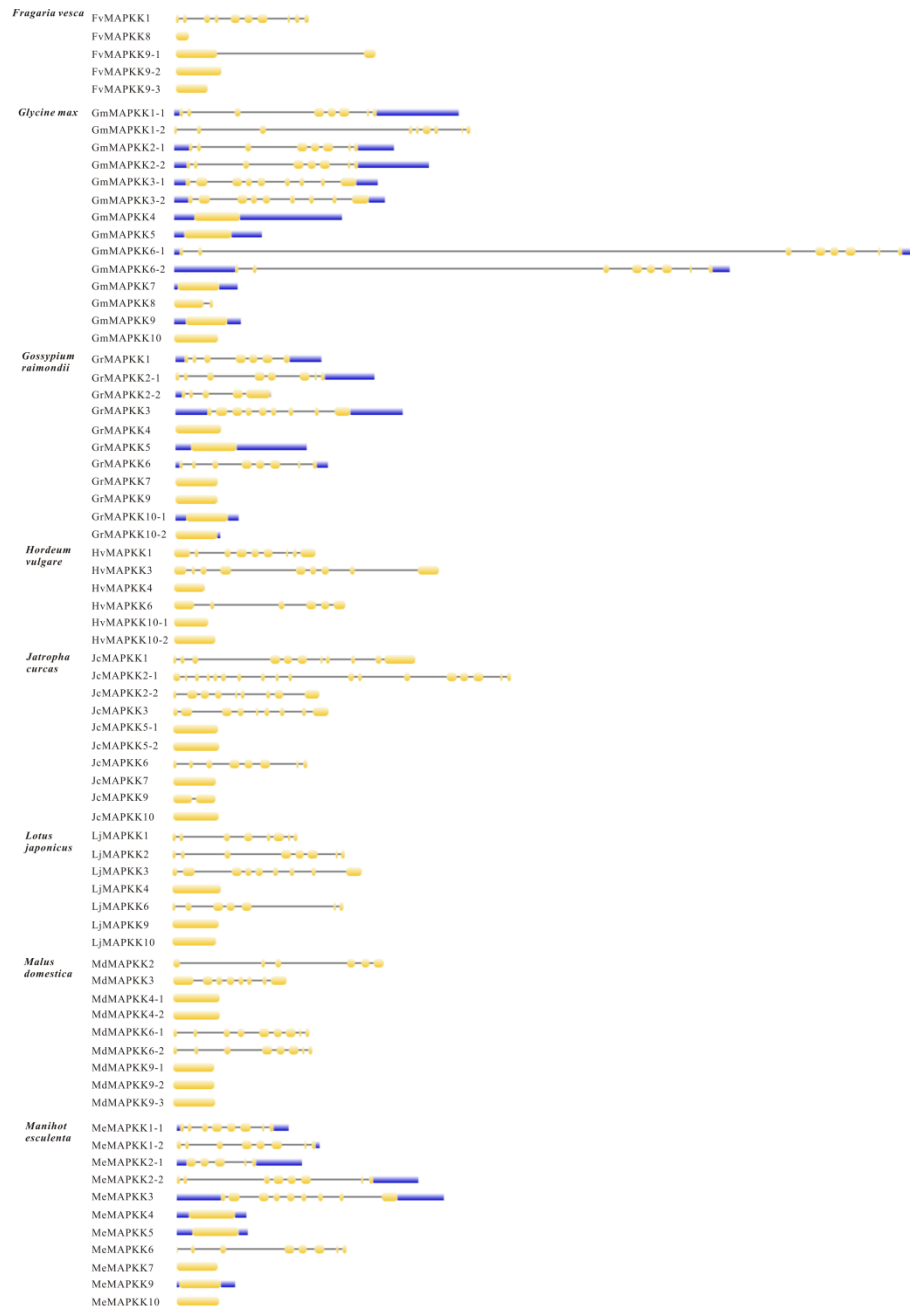

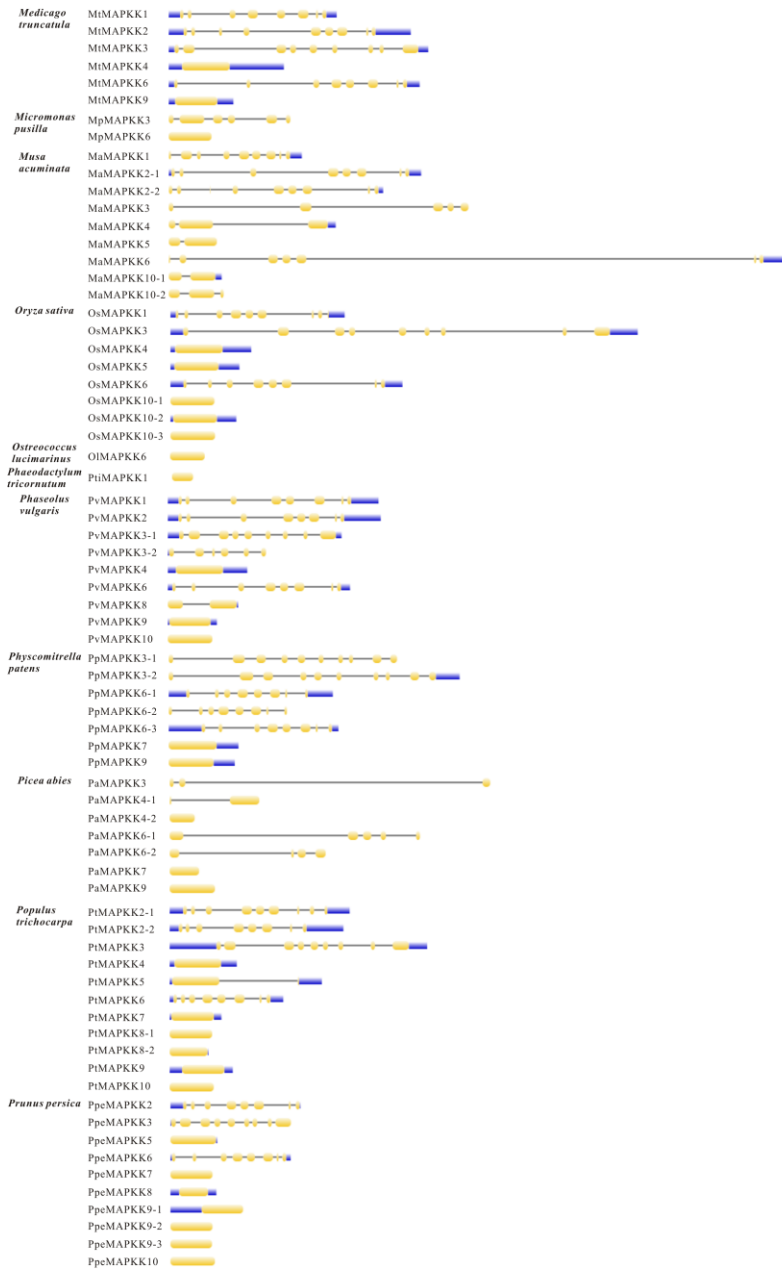

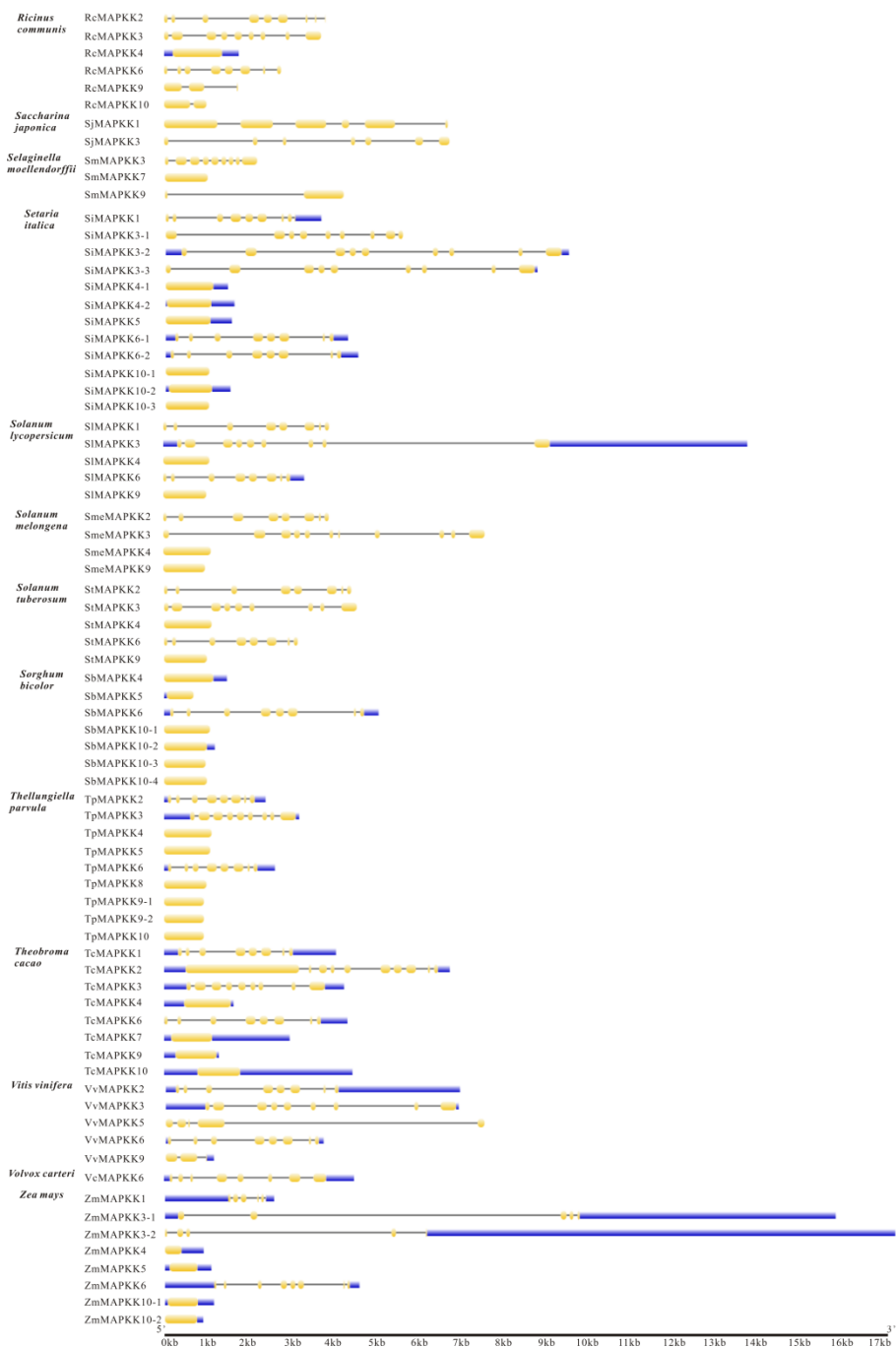

Supplement: Supplementary file 15 — Fig. S8. Maximum Likelihood phylogenetic trees of plant group B MAPKKs. (PDF 1039 kb) [file 12864_2018_4793_MOESM15_ESM.pdf]

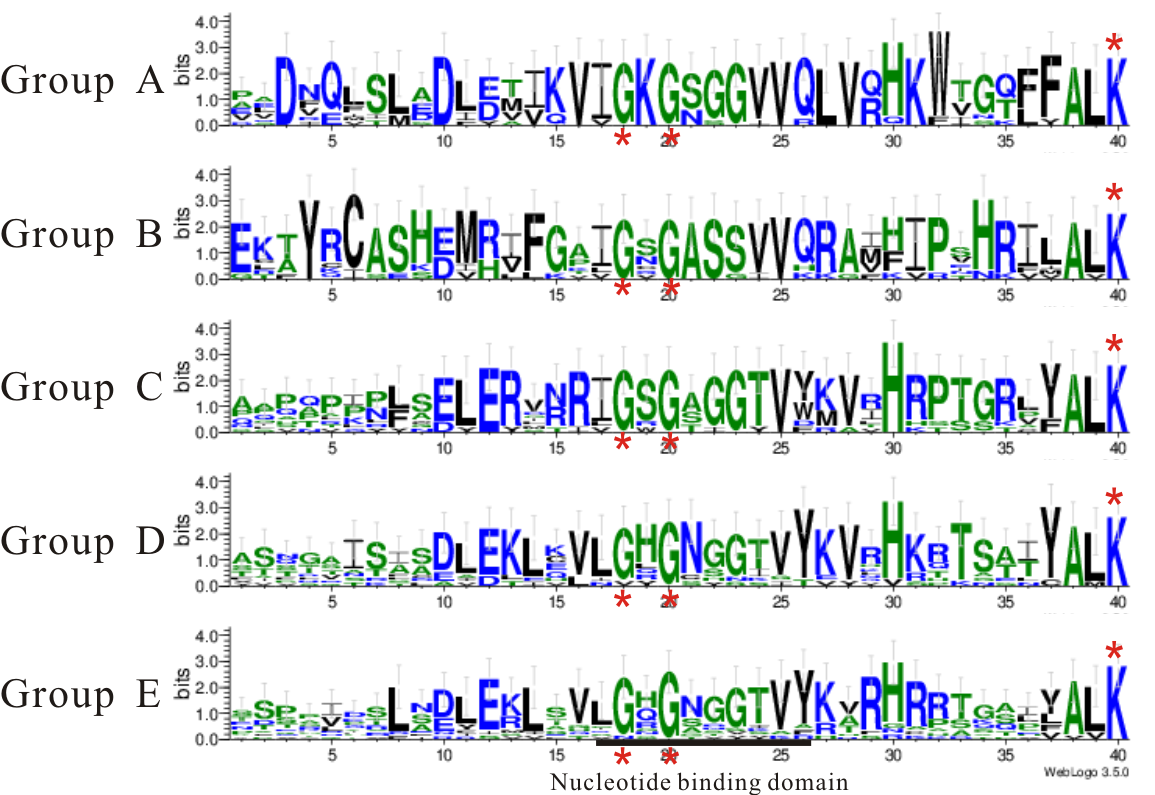

Supplement: Supplementary file 18 — Fig. S11. Syntenic proofs of plant Group C MAPKKs. (TIF 2692 kb) [file 12864_2018_4793_MOESM18_ESM.tif]

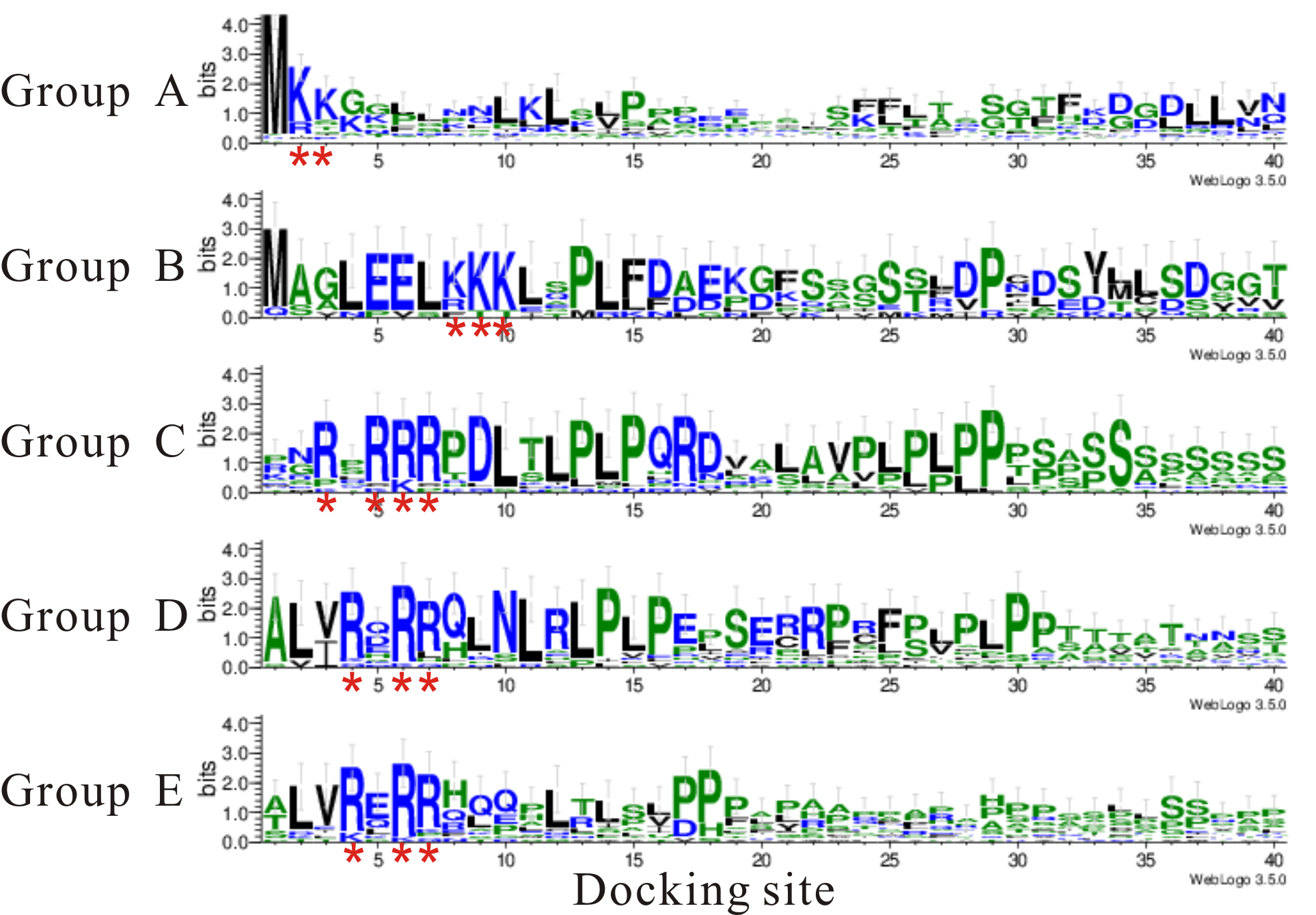

Supplement: Supplementary file 19 — Fig. S12. Expression profiles of other plant MAPKK genes. Y-axis represents RPKM value. The expression data were downloaded from rice (GSE27726), poplar (GSE30507) and maize (GSE27004) oligonucleotide array database, respectively. (TIF 7561 kb) [file 12864_2018_4793_MOESM19_ESM.tif]
