# Supplementary material for: Comparative analysis of plant MKK gene family reveals novel expansion mechanism of the members and sheds new light on functional conservation
Source: BMC Genomics. 2018 May 29;19:407. doi: 10.1186/s12864-018-4793-8 (PMC5975520; doi:10.1186/s12864-018-4793-8)
Supplement: Supplementary file 14 — Fig. S7. Syntenic proofs of Group E MAPKKs in monocots. (DOC 51 kb) [file 12864_2018_4793_MOESM14_ESM.doc]

**Table S8 A lists of the RT-qPCR primers for the *MAPKK* genes of *B. distachyon.***

| Gene name | Gene model | Forward Primer | Reward Primer |
| --- | --- | --- | --- |
| BdMKK1 | Bradi1g51000 | CAAATTCCTGACGCAGAGCG | CGAACCAGTTGCACGATTCC |
| BdMKK3-1 | Bradi4g39490 | ACATTCCAAGCAAGCGGGTA | CCAATAAGGTTCCCACCATG |
| BdMKK3-2 | Bradi1g41860 | GAGATCCATGGGGTGCTACT | AAGGTTTCCACCATGACAGC |
| BdMKK3-3 | Bradi3g11260 | ATAGCCATCGAGGAATCGC | ATGCAATGCACTTCCTCTC |
| BdMKK4 | Bradi3g53650 | CTCCAGAAGAATCCGGCGAG | CGACACGGGAATCCATCCAA |
| BdMKK5 | Bradi1g46880 | GTCAGGTCGATCGTTGGAGT | GGAGCAGAAAAGCAGGGACA |
| BdMKK6 | Bradi1g75150 | TGGCCGCTTCCCCTATACTC | CGCGGGATCCTTTTGTATG |
| BdMKK10-1 | Bradi1g11525 | GTACGCCCTCAAGGTGCAG | GGAACTGGGAGAACGCCTC |
| BdMKK10-2 | Bradi1g69400 | GGGACTTGTAGGCGAAACGA | CGCCACATGGGACAGGTTAT |
| BdMKK10-3 | Bradi1g10800 | GTGCTGTCTGACGGTGAGG | CAACAAACGGGTGAGCCAG |
| BdMKK10-5 | Bradi1g10790 | GGAGCTCAGGGGATTCATCG | CCATATTCCTCCCGGCGAC |
| BdActin | Bradi2g24070 | CCCGATGGACAGGTTATCACTA | ATAGAGCCACCAATCCAAACAC |
