# Supplementary material for: Comparative analysis of plant MKK gene family reveals novel expansion mechanism of the members and sheds new light on functional conservation
Source: BMC Genomics. 2018 May 29;19:407. doi: 10.1186/s12864-018-4793-8 (PMC5975520; doi:10.1186/s12864-018-4793-8)
Supplement: Supplementary file 16 — Fig. S9. Maximum Likelihood phylogenetic trees of plant group C MAPKKs. The red circle represents duplication events. (PDF 685 kb) [file 12864_2018_4793_MOESM16_ESM.pdf]

Table S3 Table representing molecular mass (in kDa) and isoelectric point of different MAPKK genes from 51 plant species identified during this study.

Gene Name	Locus ID	pI	Mol. Weight (kD)
Actinidia chinensis			
AcMAPKK1	Achn156001	5.28	41.354
AcMAPKK3	Achn118561	5.51	57.835
AcMAPKK4	Achn226251	8.58	37.495
AcMAPKK5-1	Achn164271	8.83	39.780
AcMAPKK5-2	Achn301271	9.07	39.884
AcMAPKK6-1	Achn069811	5.92	31.069
AcMAPKK6-2	Achn358101	6.39	50.035
AcMAPKK9	Achn036041	8.79	35.370
AcMAPKK10	Achn313401	6.06	38.636
Amborella trichopoda			
AtrMAPKK2	ATR_00146G00450	5.70	39.658
AtrMAPKK3	ATR_00083G00340	5.63	57.470
AtrMAPKK4	ATR_00011G01230	9.20	38.020
AtrMAPKK5	ATR_00011G01240	9.39	37.589
AtrMAPKK6	ATR_00056G01260	5.66	35.128
AtrMAPKK9	ATR_00176G00020	9.13	45.661
Arabidopsis thaliana			
AtMAPKK1	At4G26070	7.53	39.210
AtMAPKK2	At4G29810	6.00	39.848
AtMAPKK3	At5G40440	5.46	57.529
AtMAPKK4	At1G51660	9.46	40.117
AtMAPKK5	At3G21220	8.97	38.329
AtMAPKK6	At5G56580	5.95	39.837
AtMAPKK7	At1G18350	8.38	34.272
AtMAPKK8	At3G06230	6.15	32.521
AtMAPKK9	At1G73500	7.97	34.347
AtMAPKK10	At1G32320	8.50	34.022
Beta vulgaris			
BvMAPKK2	BV0G78470	5.29	39.530
BvMAPKK3	BV4G01230	5.16	57.841
BvMAPKK5	BV8G13900	9.09	41.423
BvMAPKK6	BV8G05680	6.56	39.850
BvMAPKK9	BV3G06820	9.00	36.158
Brachypodium distachyon			
BdMAPKK1	Bradi1g51000	5.46	38.645
BdMAPKK3-1	Bradi4g39490	5.70	58.463
BdMAPKK3-2	Bradi1g41860	5.66	58.602
BdMAPKK3-3	Bradi3g11260	5.74	63.329

BdMAPKK4	Bradi3g53650	9.47	38.428
BdMAPKK5	Bradi1g46880	9.21	36.987
BdMAPKK6	Bradi1g75150	5.52	39.964
BdMAPKK10-1	Bradi1g11525	7.65	36.875
BdMAPKK10-2	Bradi1g69400	6.56	36.090
BdMAPKK10-3	Bradi1g10800	8.39	36.520
BdMAPKK10-4	Bradi1g10770	9.07	35.921
BdMAPKK10-5	Bradi1g10790	9.29	34.734
Brassica napus			
BnMAPKK1	JQ708028	6.46	39.369
BnMAPKK2	JQ708029	5.80	40.247
BnMAPKK3	JQ708030	5.48	57.405
BnMAPKK4	JQ708031	9.33	39.294
BnMAPKK5	KC246595	9.12	36.652
BnMAPKK6	JQ708032	5.94	39.773
BnMAPKK8	XM_013841956	6.10	35.236
BnMAPKK9	JQ708033	8.53	33.890
Brassica oleracea			
BoMAPKK1	Bol042272	7.07	39.125
BoMAPKK2	Bol020940	5.59	40.249
BoMAPKK4	Bol031584	9.30	37.349
BoMAPKK5-1	Bol018796	8.40	36.170
BoMAPKK5-2	Bol026624	6.43	23.744
BoMAPKK5-3	Bol026625	5.60	22.450
BoMAPKK6-1	Bol026078	5.95	39.943
BoMAPKK6-2	Bol009929	5.91	28.833
BoMAPKK7	Bol039425	6.99	39.358
BoMAPKK9	Bol040005	7.56	34.113
Brassica rapa			
BrMAPKK1	BR03G48960	6.71	39.367
BrMAPKK2	BR01G07590	5.86	40.236
BrMAPKK3	BR04G09820	5.48	57.560
BrMAPKK4-1	BR05G17730	9.17	37.244
BrMAPKK4-2	BR08G02520	9.33	39.275
BrMAPKK5-1	BR01G29490	8.12	36.656
BrMAPKK5-2	BR03G36620	7.96	42.076
BrMAPKK5-3	BR03G36630	8.99	36.595
BrMAPKK6-1	BR03G12020	5.75	38.658
BrMAPKK6-2	BR10G09810	5.94	39.745
BrMAPKK8	BR02G34140	5.68	34.150
BrMAPKK9-1	BR02G22200	7.57	33.905
BrMAPKK9-2	BR07G20830	6.99	34.289
BrMAPKK10	BR09G26620	7.03	34.438

Capsella rubella			
CruMAPKK1-1	CRU_006G25040	8.34	44.632
CruMAPKK1-2	CRU_007G14010	8.89	41.294
CruMAPKK2	CRU_004G14330	5.68	39.837
CruMAPKK3	CRU_007G33860	5.57	57.626
CruMAPKK4	CRU_001G38610	9.38	40.347
CruMAPKK5	CRU_003G20230	9.07	37.440
CruMAPKK6	CRU_008G16640	5.95	39.903
CruMAPKK7	CRU_001G17210	8.31	34.843
CruMAPKK8	CRU_003G05430	6.61	35.452
CruMAPKK9	CRU_002G19080	8.04	34.659
CruMAPKK10	CRU_001G29220	8.31	34.736
Capsicum annuum			
CaMAPKK2	CA00G74230	5.68	39.435
CaMAPKK3	CA03G23860	5.48	41.292
CaMAPKK5	CA03G36820	8.86	41.170
CaMAPKK6	CA00G86340	5.89	41.586
CaMAPKK9	CA03G22790	8.89	36.757
Carica papaya			
CpMAPKK1	CP00371G00040	5.78	39.333
CpMAPKK3	CP00108G00610	5.44	57.709
CpMAPKK5	CP46236G00010	6.82	24.144
CpMAPKK6	CP00078G00290	6.08	39.777
CpMAPKK7	CP00003G03590	9.24	33.287
CpMAPKK9-1	CP00756G00010	5.66	16.464
CpMAPKK9-2	CP01155G00030	5.59	34.089
CpMAPKK9-3	CP01155G00040	5.56	14.926
CpMAPKK10	CP00090G00140	5.72	16.084
Chlamydomonas reinhardtii			
CrMAPKK2	CR13G00450	8.16	47.344
CrMAPKK3	CR06G00090	6.87	50.286
Citrullus lanatus			
ClMAPKK1	CL11G17340	5.40	39.394
ClMAPKK2	CL03G15020	5.26	38.126
ClMAPKK3	CL10G12060	5.53	57.766
ClMAPKK4	CL07G12000	8.91	41.445
ClMAPKK6	CL11G16940	6.27	39.734
ClMAPKK9	CL04G10340	6.23	23.601
Citrus sinensis			
CsMAPKK2	CS00112G00060	5.81	43.352
CsMAPKK3	CS00116G00190	5.77	57.772
CsMAPKK4	CS00729G00050	9.48	42.293
CsMAPKK6	CS00027G00740	5.72	40.030

CsMAPKK8	CS06934G00010	8.47	33.662
CsMAPKK9	CS00315G00070	8.55	36.234
CsMAPKK10	CS00488G00010	8.72	37.921
Coffea canephora			
CcMAPKK2	Cc06_g11750	6.15	39.214
CcMAPKK3	Cc00_g04720	5.47	57.887
CcMAPKK4	Cc04_g00500	9.24	41.669
CcMAPKK6	Cc06_g10630	6.22	63.887
CcMAPKK9	Cc04_g09350	8.69	36.495
CcMAPKK10	Cc08_g03210	6.31	37.879
Cucumis melo			
CmMAPKK1	CM00003G07700	5.38	34.199
CmMAPKK2	CM00025G02930	5.11	38.274
CmMAPKK3	CM00098G00010	5.52	57.833
CmMAPKK5	CM00083G00040	9.04	41.439
CmMAPKK6	CM00003G07280	6.60	40.576
CmMAPKK9	CM00001G02470	8.25	35.970
Cucumis sativus			
CsaMAPKK2-1	Csa1M589750.1	5.21	39.862
CsaMAPKK2-2	Csa2M000340.1	5.33	39.438
CsaMAPKK3	Csa3M839800.1	5.67	57.822
CsaMAPKK4	Csa3M651720.1	8.91	41.405
CsaMAPKK6	Csa2M000780.1	6.42	40.282
CsaMAPKK9	Csa1M042980.1	8.25	35.866
Dianthus caryophyllus			
DcMAPKK1	Dca12122.1	5.48	39.526
DcMAPKK3	Dca49240.1	5.50	56.589
DcMAPKK4	Dca4424.1	8.39	34.136
DcMAPKK5	Dca43553.1	9.01	42.567
DcMAPKK6-1	Dca32756.1	5.78	42.445
DcMAPKK6-2	Dca43468.1	5.70	29.008
DcMAPKK8	Dca52805.1	9.03	34.894
DcMAPKK10	Dca57202.1	6.26	36.623
Eucalyptus grandis			
EgMAPKK2	EG0003G06980	5.50	35.610
EgMAPKK3	EG0009G14680	5.51	58.114
EgMAPKK6	EG0003G28720	6.14	40.081
EgMAPKK7	EG0011G19010	6.75	34.977
EgMAPKK9	EG0008G04760	7.62	35.850
EgMAPKK10	EG0005G02270	5.79	36.427
Fragaria vesca			
FvMAPKK1	FV1G09520	5.79	46.023
FvMAPKK8	FV5G02850	5.54	11.276

FvMAPKK9-1	FV5G16270	8.57	44.644
FvMAPKK9-2	FV5G02870	6.01	38.866
FvMAPKK9-3	FV5G02840	6.44	26.718
Glycine max			
GmMAPKK1-1	GM15G18860	5.75	40.050
GmMAPKK1-2	GM09G07661	6.06	30.176
GmMAPKK2-1	GM13G16650	5.25	39.698
GmMAPKK2-2	GM17G06020	5.18	39.540
GmMAPKK3-1	GM05G08720	5.78	57.775
GmMAPKK3-2	GM19G00220	5.67	58.751
GmMAPKK4	GM07G00520	9.35	39.383
GmMAPKK5	GM08G23900	9.24	40.599
GmMAPKK6-1	GM10G15850	5.95	40.125
GmMAPKK6-2	GM02G32980	6.08	39.841
GmMAPKK7	GM09G30300	6.31	35.138
GmMAPKK8	GM09G30310	6.59	28.090
GmMAPKK9	GM07G11910	6.60	35.065
GmMAPKK10	GM01G01980	6.18	37.815
Gossypium raimondii			
GrMAPKK1	GR09G03380	5.49	40.085
GrMAPKK2-1	GR01G01360	6.19	40.228
GrMAPKK2-2	GR10G08530	8.71	40.910
GrMAPKK3	GR07G07560	5.62	57.653
GrMAPKK4	GR10G22180	9.30	38.875
GrMAPKK5	GR09G11780	8.92	38.889
GrMAPKK6	GR13G22960	6.28	39.932
GrMAPKK7	GR08G29170	8.40	36.347
GrMAPKK9	GR08G29180	7.63	36.226
GrMAPKK10-1	GR03G18400	7.10	35.743
GrMAPKK10-2	GR08G22510	7.09	35.424
Hordeum vulgare			
HvMAPKK1	HV135797G00010	6.14	38.383
HvMAPKK3	HV36969G00010	6.15	42.191
HvMAPKK4	HV160058G00010	9.23	37.588
HvMAPKK6	HV135110G00010	5.61	40.016
HvMAPKK10-1	HV42885G00010	9.41	12.946
HvMAPKK10-2	HV7730G00010	6.37	35.291
Jatropha curcas			
JcMAPKK1	XP_012066794	5.42	73.441
JcMAPKK2-1	Jcr4S00005.240	8.18	75.011
JcMAPKK2-2	Jcr4S07011.20	6.72	52.564
JcMAPKK3	Jcr4S00518.50	5.70	53.061
JcMAPKK5-1	Jcr4S10674.40	9.37	38.320

JcMAPKK5-2	Jcr4S26937.20	9.45	39.280
JcMAPKK6	Jcr4S01567.130	5.96	39.955
JcMAPKK7	Jcr4S00008.310	5.81	36.719
JcMAPKK9	Jcr4S08175.20	7.03	33.478
JcMAPKK10	Jcr4S12790.10	6.13	39.158
Lotus japonicus			
LjMAPKK1	chr6.CM0013.1740.r2.d	5.44	34.105
LjMAPKK2	chr4.CM0297.320.r2.m	5.33	39.781
LjMAPKK3	chr2.CM0263.270.r2.m	5.67	58.677
LjMAPKK4	chr3.LjB21L17.150.r2.a	9.27	41.075
LjMAPKK6	chr2.CM0065.640.r2.d	5.42	36.553
LjMAPKK9	LjSGA_014749.1.1	8.36	39.273
LjMAPKK10	LjSGA_023606.1	5.97	37.897
Malus domestica			
MdMAPKK2	MD02G007060	6.18	36.020
MdMAPKK3	MD09G001900	5.27	61.499
MdMAPKK4-1	MD09G012300	9.06	39.447
MdMAPKK4-2	MD17G012670	9.20	39.388
MdMAPKK6-1	MD02G005910	6.13	46.334
MdMAPKK6-2	MD15G014130	5.46	39.743
MdMAPKK9-1	MD00G332250	7.12	35.503
MdMAPKK9-2	MD16G011070	6.86	34.678
MdMAPKK9-3	MD06G019470	7.08	36.213
Manihot esculenta			
MeMAPKK1-1	ME09809G00210	6.22	40.509
MeMAPKK1-2	ME09809G00230	6.14	40.895
MeMAPKK2-1	ME07318G00190	5.46	28.344
MeMAPKK2-2	ME10493G00760	6.25	39.602
MeMAPKK3	ME05694G00070	5.83	57.533
MeMAPKK4	ME01027G00160	9.26	39.526
MeMAPKK5	ME09974G00010	9.26	39.708
MeMAPKK6	ME09501G00340	6.06	37.921
MeMAPKK7	ME10870G00010	5.93	34.958
MeMAPKK9	ME11170G00070	8.52	36.306
MeMAPKK10	ME04795G00070	6.39	36.601
Medicago truncatula			
MtMAPKK1	MT2G040510	6.26	40.097
MtMAPKK2	MT4G125800	5.19	42.212
MtMAPKK3	MT6G005210	5.39	58.035
MtMAPKK4	MT4G005830	9.39	40.450
MtMAPKK6	MT6G090470	5.76	39.855
MtMAPKK9	MT6G071280	8.02	36.030
Micromonas pusilla			

MpMAPKK3	MP14G01300	7.61	49.270
MpMAPKK6	MP06G05440	5.92	35.449
Musa acuminata			
MaMAPKK1	MA10G21630	7.92	47.351
MaMAPKK2-1	MA07G09900	6.17	38.617
MaMAPKK2-2	MA10G28080	5.94	38.732
MaMAPKK3	MA09G19160	5.79	32.589
MaMAPKK4	MA03G05070	8.85	51.105
MaMAPKK5	MA04G06010	9.76	36.191
MaMAPKK6	MA09G00100	5.60	35.239
MaMAPKK10-1	MA11G09660	6.32	31.041
MaMAPKK10-2	MA06G11530	7.11	32.837
Oryza sativa			
OsMAPKK1	Os06G05520	5.45	39.164
OsMAPKK3	Os06G27890	5.73	58.434
OsMAPKK4	Os02G54600	9.45	39.943
OsMAPKK5	Os06G09180	8.57	37.104
OsMAPKK6	Os01G32660	5.54	40.000
OsMAPKK10-1	Os02G46760	8.56	36.479
OsMAPKK10-2	Os03G12390	6.42	36.384
OsMAPKK10-3	Os03G50550	9.01	36.182
Ostreococcus lucimarinus			
OlMAPKK6	OL04G03760	5.62	29.477
Phaeodactylum tricornutum			
PtiMAPKK1	PTI_01G05340	6.64	17.867
Phaseolus vulgaris			
PvMAPKK1	Phvul.009G229800.1	5.56	39.772
PvMAPKK2	Phvul.003G134100.1	5.27	39.685
PvMAPKK3-1	Phvul.004G010400.1	5.61	57.888
PvMAPKK3-2	Phvul.007G112700.1	5.36	28.482
PvMAPKK4	Phvul.010G163000.1	9.35	40.542
PvMAPKK6	Phvul.004G174500.1	5.95	39.811
PvMAPKK8	Phvul.004G147900.1	6.46	35.987
PvMAPKK9	Phvul.004G147800.1	8.03	34.676
PvMAPKK10	Phvul.002G138400.1	6.35	38.348
Physcomitrella patens			
PpMAPKK3-1	PP00092G00080	5.85	57.697
PpMAPKK3-2	PP00151G00010	6.00	59.304
PpMAPKK6-1	PP00050G00300	8.06	39.138
PpMAPKK6-2	PP00106G00380	7.71	38.730
PpMAPKK6-3	PP00032G01020	6.32	38.952
PpMAPKK7	PP00114G00500	8.93	40.689
PpMAPKK9	PP00016G01540	8.97	38.038

Picea abies			
PaMAPKK3	MA_41206g0010	5.11	14.944
PaMAPKK4-1	MA_10192951g0010	9.06	26.695
PaMAPKK4-2	MA_10192951g0020	8.97	21.927
PaMAPKK6-1	MA_177636g0010	6.85	33.973
PaMAPKK6-2	MA_206154g0010	6.23	25.616
PaMAPKK7	MA_10372232g0010	8.89	23.688
PaMAPKK9	MA_10194g0020	9.02	38.081
Populus trichocarpa			
PtMAPKK2-1	PT18G05080	5.78	42.129
PtMAPKK2-2	PT06G14650	5.86	39.542
PtMAPKK3	PT01G34550	5.28	58.205
PtMAPKK4	PT10G24930	9.30	38.951
PtMAPKK5	PT08G00980	9.37	40.403
PtMAPKK6	PT18G06850	5.95	40.070
PtMAPKK7	PT15G03070	6.59	36.299
PtMAPKK8-1	PT08G18370	6.73	35.817
PtMAPKK8-2	PT10G04950	6.35	31.879
PtMAPKK9	PT12G04320	8.04	36.061
PtMAPKK10	PT01G13880	6.60	37.225
Prunus persica			
PpeMAPKK2	PPE_007G22450	5.60	39.537
PpeMAPKK3	PPE_003G26810	5.51	57.728
PpeMAPKK5	PPE_003G15010	9.08	39.198
PpeMAPKK6	PPE_007G23740	5.79	39.903
PpeMAPKK7	PPE_001G05740	7.12	36.117
PpeMAPKK8	PPE_005G23760	5.88	25.110
PpeMAPKK9-1	PPE_001G08120	6.51	35.750
PpeMAPKK9-2	PPE_001G18890	6.82	36.072
PpeMAPKK9-3	PPE_001G07660	6.26	35.679
PpeMAPKK10	PPE_005G03470	5.74	38.444
Ricinus communis			
RcMAPKK2	RC29912G00340	5.56	37.711
RcMAPKK3	RC29917G00620	5.78	57.420
RcMAPKK4	RC29748G00070	9.35	42.533
RcMAPKK6	RC29912G01450	5.69	39.981
RcMAPKK9	RC29929G02980	9.46	29.549
RcMAPKK10	RC30054G00130	6.63	33.320
Saccharina japonica			
SjMAPKK1	SJ16021	8.30	127.882
SjMAPKK3	SJ02374	8.14	34.422
Selaginella moellendorffii			
SmMAPKK3	SM00000G08420	5.89	56.905

SmMAPKK7	SM00011G01810	8.86	37.089
SmMAPKK9	SM00017G02550	9.03	36.540
Setaria italica			
SiMAPKK1	SI004G03650	6.36	39.064
SiMAPKK3-1	SI006G16020	8.65	52.623
SiMAPKK3-2	SI001G31910	5.72	58.415
SiMAPKK3-3	SI001G31920	5.80	58.515
SiMAPKK4-1	SI001G36010	9.45	39.551
SiMAPKK4-2	SI005G23750	8.68	37.242
SiMAPKK5	SI004G04440	9.21	37.390
SiMAPKK6-1	SI003G06250	5.57	39.954
SiMAPKK6-2	SI005G30780	5.47	39.877
SiMAPKK10-1	SI009G11680	8.48	34.638
SiMAPKK10-2	SI009G50030	6.08	35.179
SiMAPKK10-3	SI009G11700	8.76	35.187
Solanum lycopersicum			
SlMAPKK1	SL12G009020	5.58	39.670
SlMAPKK3	SL03G019850	5.49	57.501
SlMAPKK4	SL03G123800	8.87	39.766
SlMAPKK6	SL03G119490	5.89	39.634
SlMAPKK9	SL03G097920	8.70	37.492
Solanum melongena			
SmeMAPKK2	Sme2.5_01917.1_g00010.1	5.47	44.976
SmeMAPKK3	Sme2.5_00861.1_g00010.1	6.13	62.115
SmeMAPKK4	Sme2.5_09230.1_g00001.1	9.01	40.862
SmeMAPKK9	Sme2.5_00088.1_g00017.1	8.27	36.439
Solanum tuberosum			
StMAPKK2	ST12G010200	5.47	39.659
StMAPKK3	ST03G024510	5.56	57.454
StMAPKK4	ST03G034170	8.87	40.819
StMAPKK6	ST03G033030	5.89	39.634
StMAPKK9	ST03G022560	8.35	37.257
Sorghum bicolor			
SbMAPKK4	SB04g035370	9.37	42.397
SbMAPKK5	SB10G006080	5.79	22.907
SbMAPKK6	SB03g033190	5.47	39.979
SbMAPKK10-1	SB04g031130	6.97	38.699
SbMAPKK10-2	SB01g042350	6.84	35.881
SbMAPKK10-3	SB01G010180	8.70	34.565
SbMAPKK10-4	SB01g010200	7.66	35.515
Thellungiella parvula			
TpMAPKK2	TP7G27620	6.09	40.273
TpMAPKK3	TP7G01490	5.75	57.620

TpMAPKK4	TP1G38310	9.41	40.532
TpMAPKK5	TP3G19220	9.10	39.480
TpMAPKK6	TP6G18360	5.83	39.691
TpMAPKK8	TP4G05470	6.38	36.880
TpMAPKK9-1	TP5G28400	8.01	34.363
TpMAPKK9-2	TP5G28670	8.01	34.363
TpMAPKK10	TP1G28580	8.06	34.199
Theobroma cacao			
TcMAPKK1	TC0009G07910	5.73	40.035
TcMAPKK2	TC0009G07920	6.13	146.588
TcMAPKK3	TC0004G01620	5.74	57.813
TcMAPKK4	TC0006G01710	9.21	40.381
TcMAPKK6	TC0009G09420	6.60	39.885
TcMAPKK7	TC0002G26500	6.21	35.774
TcMAPKK9	TC0003G06310	8.09	35.833
TcMAPKK10	TC0003G30210	6.36	37.061
Vitis vinifera			
VvMAPKK2	VV11G03170	6.00	39.154
VvMAPKK3	VV14G11130	5.56	57.843
VvMAPKK5	VV09G01210	10.15	43.654
VvMAPKK6	VV11G04310	6.02	39.852
VvMAPKK9	VV17G07540	6.38	24.669
Volvox carteri			
VcMAPKK6	VC00006G02490	6.11	44.277
Zea mays			
ZmMAPKK1	ZM09G04800	5.43	22.661
ZmMAPKK3-1	ZM03G15310	6.64	34.239
ZmMAPKK3-2	ZM05G06160	5.06	23.913
ZmMAPKK4	ZM05G42740	9.40	38.800
ZmMAPKK5	ZM09G02500	9.36	37.635
ZmMAPKK6	ZM03G33010	5.56	39.875
ZmMAPKK10-1	ZM05G37270	7.28	40.124
ZmMAPKK10-2	ZM01G08460	6.51	42.959
Homo sapiens			
HsMAPKK1	uc010bhq.4	6.18	43.439
HsMAPKK2	uc002lzk.4	6.12	44.424
HsMAPKK3	uc002gys.4	7.05	39.318
HsMAPKK4	uc002gnj.5	8.28	44.287
HsMAPKK5	uc002aqu.4	5.98	50.111
HsMAPKK6	uc002jij.4	7	37.492
HsMAPKK7	uc002mit.4	9.26	47.485
Saccharomyces cerevisiae			
ScPBS2	YJL128C	9.44	72.72

ScMAPKK1	YOR231W	8.32	56.72
ScMAPKK2	YPL140C	9.1	56.757
ScSTE7	YDL159W	9.92	57.708
Schizosaccharomyces pombe			
SpWis1	SPBC409.07c	9.4	64.76
SpByr1	SPAC1D4.13	7.52	38.19
SpPek1	SPBC543.07	8.2	40.71
