# Supplementary material for: Comparative analysis of plant MKK gene family reveals novel expansion mechanism of the members and sheds new light on functional conservation
Source: BMC Genomics. 2018 May 29;19:407. doi: 10.1186/s12864-018-4793-8 (PMC5975520; doi:10.1186/s12864-018-4793-8)
Supplement: Supplementary file 17 — Fig. S10. Maximum Likelihood phylogenetic trees of plant group D MAPKKs. The red circle represents duplication events. (PDF 148 kb) [file 12864_2018_4793_MOESM17_ESM.pdf]

Table S4 Table showing average amino acid composition of plant MAPKKs

Amino Acids	Average amino acid composition of MAPKK gene
Ala	6.51
Cys	2.17
Asp	5.33
Glu	5.71
Phe	4.01
Gly	7.03
His	2.98
Ile	5.85
Lys	4.98
Leu	10.38
Met	2.37
Asn	3.70
Pro	6.35
Gln	3.98
Arg	5.48
Ser	8.70
Thr	4.16
Val	6.35
Trp	0.85
Tyr	3.11
