# Supplementary material for: Comparative analysis of plant MKK gene family reveals novel expansion mechanism of the members and sheds new light on functional conservation
Source: BMC Genomics. 2018 May 29;19:407. doi: 10.1186/s12864-018-4793-8 (PMC5975520; doi:10.1186/s12864-018-4793-8)
Supplement: Supplementary file 20 — Fig. S13. Expression profiles of B. distachyon MAPKK genes in different tissues and UV-B signaling. (DOCX 15 kb) [file 12864_2018_4793_MOESM20_ESM.docx]

**Table S5** Molecular evolutionary analysis of the *MAPKK* genes in different motif.

| Motif | Group | N | Ka | Ks | ώ | G+C content |
| --- | --- | --- | --- | --- | --- | --- |
| D(I/L/V)K motif and S/T-X5-S/T motif | A | 122 | 0.14972 | 0.77515 | 0.193 | 0.411 |
|  | A＊ | 106 | 0.14758 | 0.77361 | 0.191 | 0.412 |
|  | B | 51 | 0.08417 | 0.66224 | 0.127 | 0.416 |
|  | B＊ | 41 | 0.07372 | 0.66339 | 0.111 | 0.417 |
|  | C | 69 | 0.1124 | 0.80132 | 0.14 | 0.491 |
|  | C＊ | 56 | 0.10506 | 0.81193 | 0.129 | 0.455 |
|  | D | 72 | 0.28047 | 0.72783 | 0.385 | 0.452 |
|  | E | 39 | 0.246 | 0.75192 | 0.327 | 0.558 |
|  | E^☆^ | 19 | 0.21283 | 0.36472 | 0.584 | 0.7 |
| D-site | A | 113 | 0.60954 | 0.58394 | 1.044 | 0.476 |
|  | A＊ | 99 | 0.60068 | 0.58402 | 1.029 | 0.46 |
|  | B | 48 | 0.24588 | 0.63819 | 0.385 | 0.45 |
|  | B＊ | 41 | 0.23773 | 0.61686 | 0.385 | 0.44 |
|  | C | 62 | 0.38128 | 0.32244 | 1.182 | 0.657 |
|  | C＊ | 51 | 0.38369 | 0.32691 | 1.174 | 0.627 |
|  | D | 68 | 0.21961 | 0.50045 | 0.439 | 0.591 |
|  | E | 39 | 0.40747 | 0.7341 | 0.555 | 0.61 |
|  | E^☆^ | 19 | 0.46202 | 0.62962 | 0.734 | 0.725 |
| NB domain and ATP binding site | A | 115 | 0.27106 | 0.52678 | 0.515 | 0.428 |
|  | A＊ | 100 | 0.26943 | 0.5172 | 0.521 | 0.423 |
|  | B | 48 | 0.12913 | 0.68805 | 0.188 | 0.453 |
|  | B＊ | 39 | 0.10577 | 0.70001 | 0.151 | 0.459 |
|  | C | 62 | 0.37593 | 0.23865 | 1.575 | 0.609 |
|  | C＊ | 51 | 0.3578 | 0.21644 | 1.653 | 0.571 |
|  | D | 69 | 0.29396 | 0.41307 | 0.712 | 0.526 |
|  | E | 39 | 0.23876 | 0.68265 | 0.35 | 0.587 |
|  | A | 115 | 0.27106 | 0.52678 | 0.515 | 0.428 |

N, number of sequences; Ka, the number of nonsynonymous substitutions per nonsynonymous site; Ks, the number of synonymous substitutions per synonymous site; ώ, Ka/Ks. ^☆^ Sequences from dicots were excluded. ＊Sequences from monocots were excluded.
